# A Meta-Analysis of Thyroid-Related Traits Reveals Novel Loci and Gender-Specific Differences in the Regulation of Thyroid Function

**DOI:** 10.1371/journal.pgen.1003266

**Published:** 2013-02-07

**Authors:** Eleonora Porcu, Marco Medici, Giorgio Pistis, Claudia B. Volpato, Scott G. Wilson, Anne R. Cappola, Steffan D. Bos, Joris Deelen, Martin den Heijer, Rachel M. Freathy, Jari Lahti, Chunyu Liu, Lorna M. Lopez, Ilja M. Nolte, Jeffrey R. O'Connell, Toshiko Tanaka, Stella Trompet, Alice Arnold, Stefania Bandinelli, Marian Beekman, Stefan Böhringer, Suzanne J. Brown, Brendan M. Buckley, Clara Camaschella, Anton J. M. de Craen, Gail Davies, Marieke C. H. de Visser, Ian Ford, Tom Forsen, Timothy M. Frayling, Laura Fugazzola, Martin Gögele, Andrew T. Hattersley, Ad R. Hermus, Albert Hofman, Jeanine J. Houwing-Duistermaat, Richard A. Jensen, Eero Kajantie, Margreet Kloppenburg, Ee M. Lim, Corrado Masciullo, Stefano Mariotti, Cosetta Minelli, Braxton D. Mitchell, Ramaiah Nagaraja, Romana T. Netea-Maier, Aarno Palotie, Luca Persani, Maria G. Piras, Bruce M. Psaty, Katri Räikkönen, J. Brent Richards, Fernando Rivadeneira, Cinzia Sala, Mona M. Sabra, Naveed Sattar, Beverley M. Shields, Nicole Soranzo, John M. Starr, David J. Stott, Fred C. G. J. Sweep, Gianluca Usala, Melanie M. van der Klauw, Diana van Heemst, Alies van Mullem, Sita H.Vermeulen, W. Edward Visser, John P. Walsh, Rudi G. J. Westendorp, Elisabeth Widen, Guangju Zhai, Francesco Cucca, Ian J. Deary, Johan G. Eriksson, Luigi Ferrucci, Caroline S. Fox, J. Wouter Jukema, Lambertus A. Kiemeney, Peter P. Pramstaller, David Schlessinger, Alan R. Shuldiner, Eline P. Slagboom, André G. Uitterlinden, Bijay Vaidya, Theo J. Visser, Bruce H. R. Wolffenbuttel, Ingrid Meulenbelt, Jerome I. Rotter, Tim D. Spector, Andrew A. Hicks, Daniela Toniolo, Serena Sanna, Robin P. Peeters, Silvia Naitza

**Affiliations:** 1 Istituto di Ricerca Genetica e Biomedica (IRGB), Consiglio Nazionale delle Ricerche, c/o Cittadella Universitaria di Monserrato, Monserrato, Cagliari, Italy; 2 Dipartimento di Scienze Biomediche, Università di Sassari, Sassari, Italy; 3 Department of Internal Medicine, Erasmus MC, Rotterdam, The Netherlands; 4 Division of Genetics and Cell Biology, San Raffaele Research Institute, Milano, Italy; 5 Università degli Studi di Trieste, Trieste, Italy; 6 Center for Biomedicine, European Academy Bozen/Bolzano (EURAC), Bolzano, Italy (Affiliated Institute of the University of Lübeck, Lübeck, Germany); 7 Department of Endocrinology and Diabetes, Sir Charles Gairdner Hospital, Nedlands, Western Australia, Australia; 8 Department of Twin Research and Genetic Epidemiology, King's College London, London, United Kingdom; 9 School of Medicine and Pharmacology, University of Western Australia, Crawley, Western Australia, Australia; 10 University of Pennsylvania School of Medicine, Philadelphia, Pennsylvania, United States of America; 11 Leiden University Medical Center, Molecular Epidemiology, Leiden, The Netherlands; 12 Netherlands Consortium for Healthy Ageing, Leiden, The Netherlands; 13 Department of Endocrinology, Radboud University Nijmegen Medical Centre, Nijmegen, The Netherlands; 14 Department of Internal Medicine, Free University Medical Center, Amsterdam, The Netherlands; 15 Genetics of Complex Traits, Peninsula College of Medicine and Dentistry, University of Exeter, Exeter, United Kingdom; 16 Institute of Behavioural Sciences, University of Helsinki, Helsinki, Finland; 17 Center for Population Studies, National Heart, Lung, and Blood Institute, Framingham, Massachusetts, United States of America; 18 Centre for Cognitive Ageing and Cognitive Epidemiology, University of Edinburgh, Edinburgh, United Kingdom; 19 Department of Psychology, University of Edinburgh, Edinburgh, United Kingdom; 20 Unit of Genetic Epidemiology and Bioinformatics, Department of Epidemiology, University Medical Center Groningen, University of Groningen, Groningen, The Netherlands; 21 Department of Medicine, University of Maryland Medical School, Baltimore, Maryland, United States of America; 22 Clinical Research Branch, National Institute on Aging, Baltimore, Maryland, United States of America; 23 Department of Cardiology, Leiden University Medical Center, Leiden, The Netherlands; 24 Department of Gerontology and Geriatrics, Leiden University Medical Center, Leiden, The Netherlands; 25 Cardiovascular Health Research Unit and Department of Medicine, University of Washington, Seattle, Washington, United States of America; 26 Geriatric Unit, Azienda Sanitaria Firenze (ASF), Florence, Italy; 27 Leiden University Medical Center, Medical Statistics and Bioinformatics, Leiden, The Netherlands; 28 Department of Pharmacology and Therapeutics, University College Cork, Cork, Ireland; 29 Vita e Salute University, San Raffaele Scientific Institute, Milano, Italy; 30 Department for Health Evidence, Radboud University Medical Centre, Nijmegen, The Netherlands; 31 Robertson Center for Biostatistics, University of Glasgow, Glasgow, United Kingdom; 32 Department of Chronic Disease Prevention, National Institute for Health and Welfare, Helsinki, Finland; 33 Department of General Practice and Primary Health Care, University of Helsinki, Helsinki, Finland; 34 Helsinki University Central Hospital, Unit of General Practice, Helsinki, Finland; 35 Vaasa Health Care Centre, Diabetes Unit, Vaasa, Finland; 36 Endocrine Unit, Fondazione Ca' Granda Policlinico and Department of Clinical Sciences and Community Health, University of Milan, Milano, Italy; 37 Peninsula NIHR Clinical Research Facility, Peninsula College of Medicine and Dentistry, University of Exeter, Exeter, United Kingdom; 38 Department of Epidemiology, Erasmus MC, Rotterdam, The Netherlands; 39 Netherlands Genomics Initiative (NGI)–sponsored Netherlands Consortium for Healthy Aging (NCHA), Rotterdam, The Netherlands; 40 Hospital for Children and Adolescents, Helsinki University Central Hospital and University of Helsinki, Helsinki, Finland; 41 Department of Clinical Epidemiology and Rheumatology, Leiden University Medical Center, Leiden, The Netherlands; 42 Pathwest Laboratory Medicine WA, Nedlands, Western Australia, Australia; 43 Dipartimento di Scienze Mediche, Università di Cagliari, c/o Cittadella Universitaria di Monserrato, Monserrato, Cagliari, Italy; 44 Laboratory of Genetics, National Institute on Aging, Baltimore, Maryland, United States of America; 45 Wellcome Trust Sanger Institute, Wellcome Trust Genome Campus, Cambridge, United Kingdom; 46 Institute for Molecular Medicine Finland (FIMM), University of Helsinki, Helsinki, Finland; 47 Department of Medical Genetics, University of Helsinki and University Central Hospital, Helsinki, Finland; 48 Department of Clinical Sciences and Community Health, University of Milan, Milano, Italy; 49 Division of Endocrinology and Metabolic Diseases, IRCCS Ospedale San Luca, Milan, Italy; 50 Cardiovascular Health Research Unit, Departments of Medicine, Epidemiology, and Health Services, University of Washington, Seattle, Washington, United States of America; 51 Group Health Research Institute, Group Health Cooperative, Seattle, Washington, United States of America; 52 Department of Medicine, Jewish General Hospital, McGill University, Montréal, Québec, Canada; 53 Departments of Human Genetics, Epidemiology, and Biostatistics, Jewish General Hospital, Lady Davis Institute, McGill University, Montréal, Québec; 54 Memorial Sloan Kettering Cancer Center, Medicine-Endocrinology, New York, New York, United States of America; 55 BHF Glasgow Cardiovascular Research Centre, Faculty of Medicine, Glasgow, United Kingdom; 56 Alzheimer Scotland Dementia Research Centre, University of Edinburgh, Edinburgh, United Kingdom; 57 Academic Section of Geriatric Medicine, Faculty of Medicine, University of Glasgow, Glasgow, United Kingdom; 58 Department of Laboratory Medicine, Radboud University Nijmegen Medical Center, Nijmegen, The Netherlands; 59 LifeLines Cohort Study, University Medical Center Groningen, University of Groningen, Groningen, The Netherlands; 60 Department of Endocrinology, University Medical Center Groningen, University of Groningen, Groningen, The Netherlands; 61 Leiden University Medical Center, Gerontology and Geriatrics, Leiden, The Netherlands; 62 Discipline of Genetics, Faculty of Medicine, Memorial University of Newfoundland, St. Johns, Newfoundland, Canada; 63 Folkhalsan Research Centre, Helsinki, Finland; 64 Vasa Central Hospital, Vasa, Finland; 65 Division of Intramural Research, National Heart, Lung, and Blood Institute, Framingham, Massachusetts, United States of America; 66 Division of Endocrinology, Hypertension, and Metabolism, Brigham and Women's Hospital and Harvard Medical School, Boston, Massachusetts, United States of America; 67 Durrer Center for Cardiogenetic Research, Amsterdam, The Netherlands; 68 Interuniversity Cardiology Institute of the Netherlands, Utrecht, The Netherlands; 69 Department of Urology, Radboud University Medical Centre, Nijmegen, The Netherlands; 70 Department of Neurology, General Central Hospital, Bolzano, Italy; 71 Department of Neurology, University of Lübeck, Lübeck, Germany; 72 Geriatric Research and Education Clinical Center, Veterans Administration Medical Center, Baltimore, Maryland, United States of America; 73 Diabetes, Endocrinology and Vascular Health Centre, Royal Devon and Exeter NHS Foundation Trust, Exeter, United Kingdom; 74 Medical Genetics Institute, Cedars-Sinai Medical Center, Los Angeles, California, United States of America; 75 Institute of Molecular Genetics–CNR, Pavia, Italy; University of Oxford, United Kingdom

## Abstract

Thyroid hormone is essential for normal metabolism and development, and overt abnormalities in thyroid function lead to common endocrine disorders affecting approximately 10% of individuals over their life span. In addition, even mild alterations in thyroid function are associated with weight changes, atrial fibrillation, osteoporosis, and psychiatric disorders. To identify novel variants underlying thyroid function, we performed a large meta-analysis of genome-wide association studies for serum levels of the highly heritable thyroid function markers TSH and FT4, in up to 26,420 and 17,520 euthyroid subjects, respectively. Here we report 26 independent associations, including several novel loci for TSH (*PDE10A*, *VEGFA*, *IGFBP5*, *NFIA*, *SOX9, PRDM11, FGF7*, *INSR*, *ABO*, *MIR1179*, *NRG1*, *MBIP*, *ITPK1*, *SASH1, GLIS3*) and FT4 (*LHX3*, *FOXE1, AADAT, NETO1/FBXO15, LPCAT2/CAPNS2*). Notably, only limited overlap was detected between TSH and FT4 associated signals, in spite of the feedback regulation of their circulating levels by the hypothalamic-pituitary-thyroid axis. Five of the reported loci (*PDE8B*, *PDE10A, MAF/LOC440389, NETO1/FBXO15*, and *LPCAT2/CAPNS2*) show strong gender-specific differences, which offer clues for the known sexual dimorphism in thyroid function and related pathologies. Importantly, the TSH-associated loci contribute not only to variation within the normal range, but also to TSH values outside the reference range, suggesting that they may be involved in thyroid dysfunction. Overall, our findings explain, respectively, 5.64% and 2.30% of total TSH and FT4 trait variance, and they improve the current knowledge of the regulation of hypothalamic-pituitary-thyroid axis function and the consequences of genetic variation for hypo- or hyperthyroidism.

## Author Summary

Levels of thyroid hormones are tightly regulated by TSH produced in the pituitary, and even mild alterations in their concentrations are strong indicators of thyroid pathologies, which are very common worldwide. To identify common genetic variants associated with the highly heritable markers of thyroid function, TSH and FT4, we conducted a meta-analysis of genome-wide association studies in 26,420 and 17,520 individuals, respectively, of European ancestry with normal thyroid function. Our analysis identified 26 independent genetic variants regulating these traits, several of which are new, and confirmed previously detected polymorphisms affecting TSH (within the *PDE8B* gene and near *CAPZB, MAF/LOC440389*, and *NR3C2*) and FT4 (within *DIO1*) levels. Gender-specific differences in the genetic effects of several variants for TSH and FT4 levels were identified at several loci, which offer clues to understand the known sexual dimorphism in thyroid function and pathology. Of particular clinical interest, we show that TSH-associated loci contribute not only to normal variation, but also to TSH values outside reference range, suggesting that they may be involved in thyroid dysfunction. Overall, our findings add to the developing landscape of the regulation of thyroid homeostasis and the consequences of genetic variation for thyroid related diseases.

## Introduction

Through the production of thyroid hormone (TH), the thyroid is essential for normal development, growth and metabolism of virtually all human tissues. Its critical role in heart, brain, bone, and general metabolism is illustrated by the clinical manifestations of thyroid disease, which affects up to 10% of the population. Low thyroid function (i.e., hypothyroidism) can lead to weight gain, high cholesterol, cognitive dysfunction, depression, and cold intolerance, whereas hyperthyroidism may result in weight loss, tachycardia, atrial fibrillation, and osteoporosis. Mild variation in thyroid function, both subclinical and within the normal range, is associated with these TH-related clinical outcomes as well [1]–[4].

The thyroid gland secretes predominantly the pro-hormone thyroxine (T4), which is converted into the active form triiodothyronine (T3) in peripheral tissues. The production of TH by the thyroid gland is regulated by the hypothalamus-pituitary-thyroid (HPT) axis, via a so-called negative feedback loop. Briefly, low levels of serum TH in hypothyroidism result in an increased release of thyroid stimulating hormone (TSH) by the pituitary, under the influence of hypothalamic thyrotropin releasing hormone (TRH) [5]. TSH, a key regulator of thyroid function, stimulates the synthesis and secretion of TH by the thyroid. When circulating TH levels are high, as in hyperthyroidism, TRH and TSH synthesis and secretion are inhibited.

In healthy (euthyroid) individuals, TSH and free T4 (FT4) levels vary over a narrower range than the broad inter-individual variation seen in the general population, suggesting that each person has a unique HPT axis set-point that lies within the population reference range [6]. Besides environmental factors such as diet, smoking and medication, little is known about the factors that influence this inter-individual variation in TSH and FT4 levels [7]–[9]. The heritability of TSH and FT4 has been estimated from twin and family studies at about 65% and 40%, respectively [10]–[12]. However, the underlying genetic variants are not fully established, and the contribution of those discovered so far to the overall variance is modest. Single nucleotide polymorphisms (SNPs) in the *phosphodiesterase type 8B* (*PDE8B*), upstream of the *capping protein (actin filament) muscle Z-line, β* (*CAPZB*) and, more recently, of the *nuclear receptor subfamily 3, group C, member 2* (*NR3C2*) and of *v-maf musculoaponeurotic fibrosarcoma oncogene homolog* (*MAF*/*LOC440389*) genes have been implicated in TSH variation by genome-wide association studies (GWAS) [13]–[15], whereas SNPs in the *iodothyronine deiodinase DIO1* have been associated with circulating levels of TH by candidate gene analysis [16]–[18].

To identify additional common variants associated with thyroid function, we performed a meta-analysis of genome-wide association data in 26,420 euthyroid individuals phenotyped for serum TSH and 17,520 for FT4 levels, respectively. In addition, we also assessed gender-specific effects and correlation with subclinical thyroid dysfunction.

## Results

To identify common genetic variants associated with serum TSH and FT4 levels, we carried out a meta-analysis of genome-wide association results from 18 studies for TSH and 15 studies for FT4 levels, which assessed the additive effect of ∼2.5 million genotyped and HapMap-imputed SNPs in relation to those traits in individuals of European ancestry (for cohort description see Table 1 and Table S1). In order to avoid bias due to the presence of thyroid pathologies, prior to analysis we excluded all individuals with TSH values outside the normal range (TSH<0.4 mIU/L and TSH>4.0 mIU/L) and those taking thyroid medication for known thyroid pathologies whenever the relevant information was available. Our meta-analysis was thereby carried out in up to 26,420 and 17,520 euthyroid subjects, respectively for TSH and FT4. Additional exclusion criteria used by individual cohorts are detailed in Table S1.

**Table 1:** Descriptive statistics of all cohorts.

Cohort	Subjects (N)	Age, mean (SD)	Age (range)	Men (%)	TSH, mean (SD)	FT4, mean (SD)
**BLSA**	593	69.9 (15.4)	22–98	54.8	2.1 (0.9)	1.1 (0.2)
**CHS**	1,655	74.6 (4.9)	67–94	41.8	2.1 (0.9)	1.2 (0.2)
**FHS**	2,140	47.4 (10.0)	21–77	49.9	1.6 (0.8)	NA
**GARP**	290	60.3 (7.5)	42–79	20.3	1.9 (0.8)	1.2 (0.2)
**HBCS**	454	60.9 (2.8)	56–68	51.3	1.8 (0.8)	1.1 (0.1)
**InChianti**	951	68.4 (15.4)	21–102	45.3	1.5 (0.8)	1.4 (0.3)
**LBC1921**	401	79.0 (0.6)	77–80	44.0	1.7 (0.8)	1.1 (0.2)
**LBC1936**	834	69.5 (0.8)	67–71	54.0	1.7 (0.8)	1.2 (0.2)
**LifeLines**	1,306	45.0 (10.0)	20–79	44.6	2.7 (4.1)	1.3 (0.2)
**LLS**	736	59.1 (6.8)	30–75	45.9	1.7 (0.8)	1.2 (0.2)
**MICROS**	1,047	44.6 (16.5)	8–94	45.7	1.9 (0.9)	1.0 (0.2)
**NBS**	1,617	61.5 (10.3)	27–78	50.7	1.6 (1.1)	1.1 (0.2)
**OOA**	1,025	49.9 (16.7)	20–97	57.8	2.2 (0.5)	NA
**PROSPER**	4,402	75.3 (3.4)	69–83	49.1	1.9 (0.8)	1.3 (0.2)
**RS**	1,346	68.7 (7.4)	55–93	40.7	1.6 (0.8)	1.3 (0.2)
**SardiNIA**	4,087	42.5 (17.7)	14–101	46.9	1.7 (0.8)	1.3 (0.2)
**TwinsUK**	2,133	46.6 (12.5)	18–82	0	1.4 (0.7)	1.1 (0.1)
**ValBorbera**	1,403	53.6 (18.3)	18–102	46.9	1.5 (0.8)	NA

The table shows descriptive statistics of all cohorts included in the meta-analysis. TSH is reported in mIU/L and FT4 in ng/dl. SD, standard deviation.

**Figure 1:**
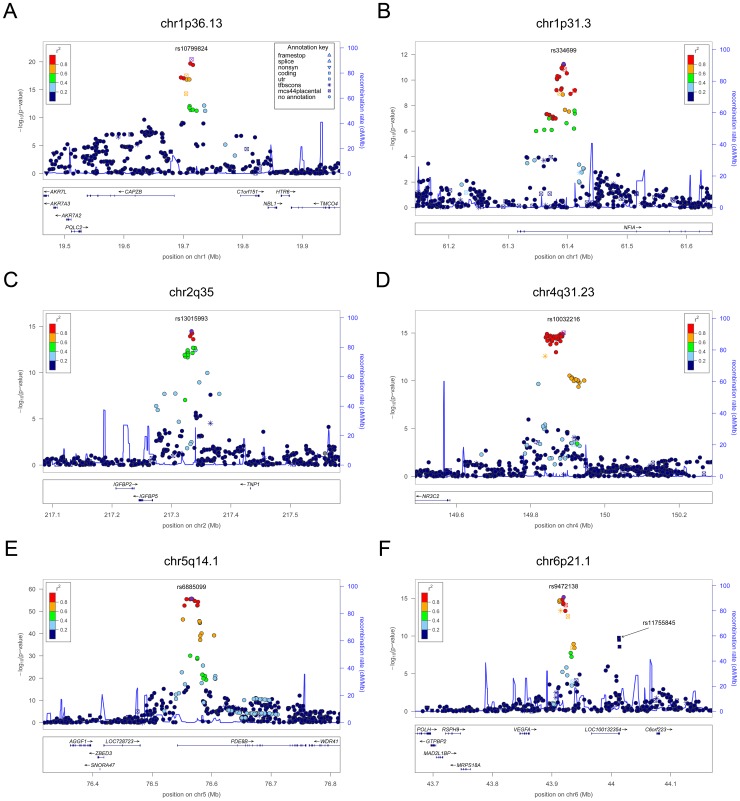
Regional association plots showing genome-wide significant loci for serum TSH. In each panel (A–F), the most significant SNP is indicated (purple circle). In panel F, an independent signal at the associated locus is indicated with an arrow. The SNPs surrounding the most significant SNP are color-coded to reflect their LD with this SNP as in the inset (taken from pairwise r^2^ values from the HapMap CEU database build 36/hg18). Symbols reflect genomic functional annotation, as indicated in the legend [61]. Genes and the position of exons, as well as the direction of transcription, are noted in lower boxes. In each panel the scale bar on the Y-axis changes according to the strength of the association.

**Figure 2:**
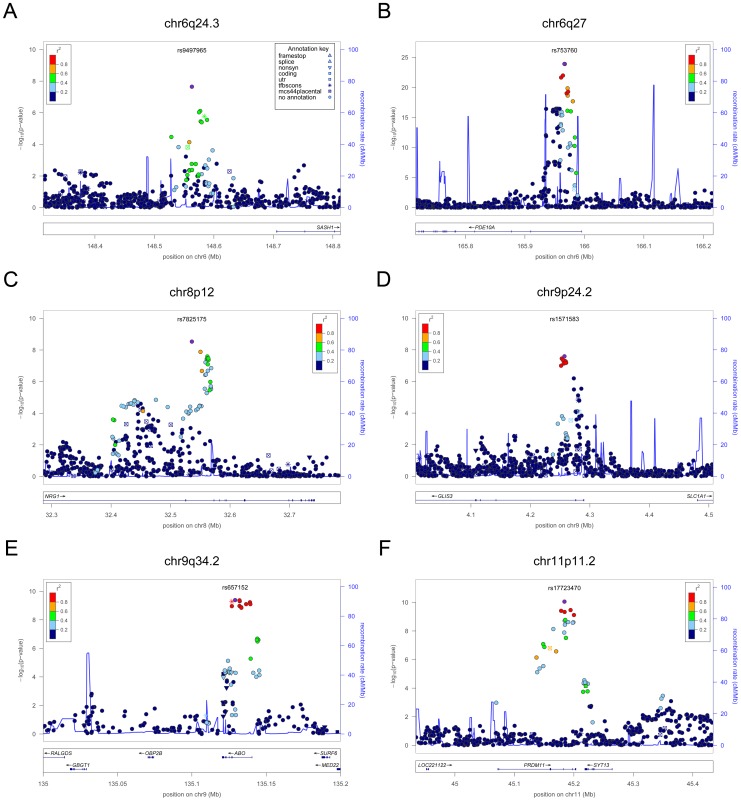
Regional association plots showing genome-wide significant loci for serum TSH. In each panel (A–F), the most significant SNP is indicated (purple circle). The SNPs surrounding the most significant SNP are color-coded to reflect their LD with this SNP as in the inset (taken from pairwise r^2^ values from the HapMap CEU database build 36/hg18). Symbols reflect genomic functional annotation, as indicated in the legend [61]. Genes and the position of exons, as well as the direction of transcription, are noted in lower boxes. In each panel the scale bar on the Y-axis changes according to the strength of the association.

**Figure 3:**
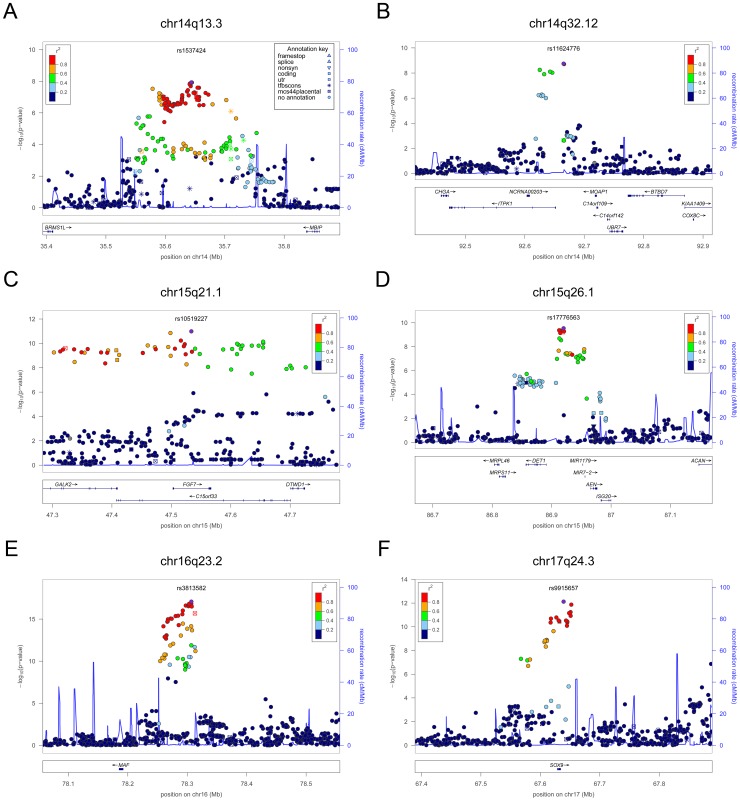
Regional association plots showing genome-wide significant loci for serum TSH. In each panel (A–F), the most significant SNP is indicated (purple circle). The SNPs surrounding the most significant SNP are color-coded to reflect their LD with this SNP as in the inset (taken from pairwise r^2^ values from the HapMap CEU database build 36/hg18). Symbols reflect genomic functional annotation, as indicated in the legend [61]. Genes and the position of exons, as well as the direction of transcription, are noted in lower boxes. In each panel the scale bar on the Y-axis changes according to the strength of the association.

**Figure 4:**
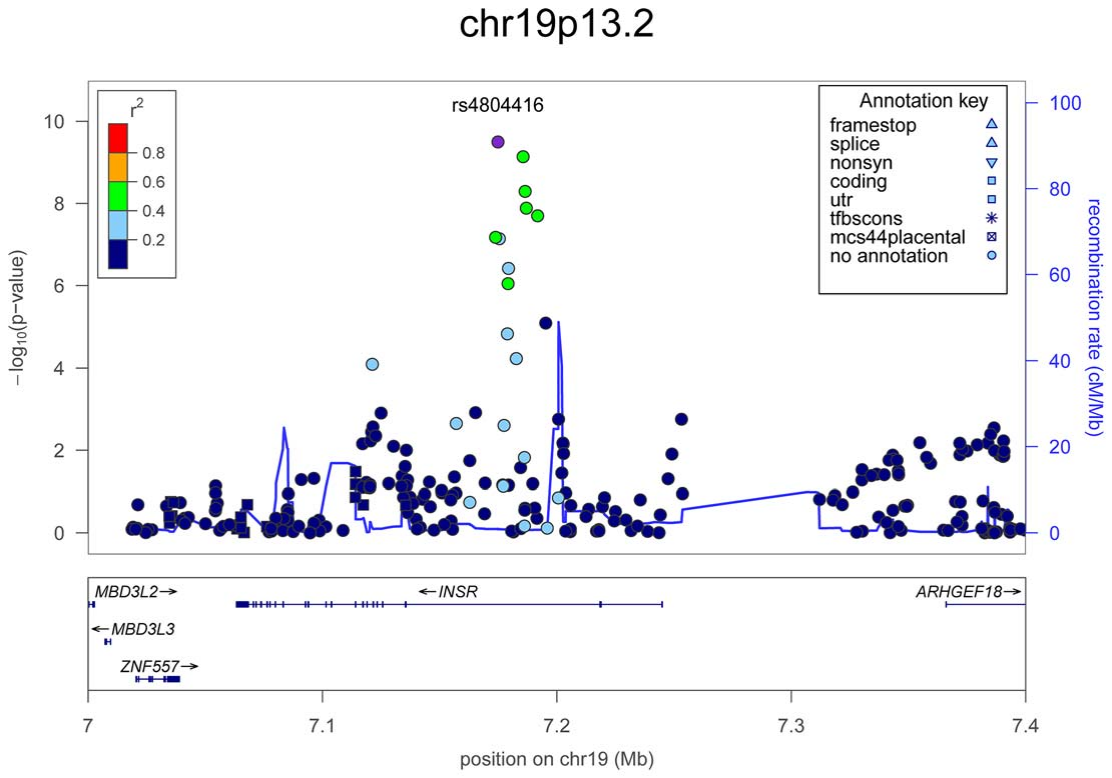
Regional association plots showing genome-wide significant loci for serum TSH. In the upper panel, the most significant SNP is indicated (purple circle). The SNPs surrounding the most significant SNP are color-coded to reflect their LD with this SNP as in the inset (taken from pairwise r^2^ values from the HapMap CEU database build 36/hg18). Symbols reflect genomic functional annotation, as indicated in the legend [61]. Genes and the position of exons, as well as the direction of transcription, are noted in the lower box. The scale bar on the Y-axis changes according to the strength of the association.

**Figure 5:**
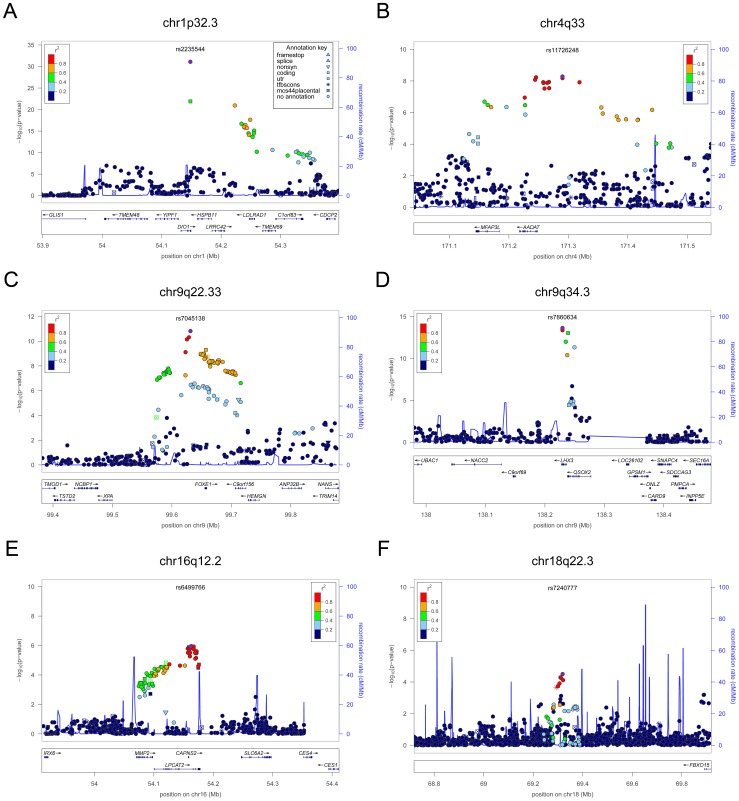
Regional association plots showing genome-wide significant loci for serum FT4. In each panel (A–F), the most significant SNP is indicated (purple circle). The SNPs surrounding the most significant SNP are color-coded to reflect their LD with this SNP as in the inset (taken from pairwise r^2^ values from the HapMap CEU database build 36/hg18). Symbols reflect genomic functional annotation, as indicated in the legend [61]. Genes and the position of exons, as well as the direction of transcription, are noted in lower boxes. In each panel the scale bar on the Y-axis changes according to the strength of the association.

Using the standard genome-wide threshold of 5×10^−8^, we observed significant associations for SNPs at 23 loci, of which 19 were associated with TSH, and 4 with FT4 (Figure S1). The results are presented in Table 2 and Figure 1, Figure 2, Figure 3, Figure 4, Figure 5. In Table S2 single cohort results for each GW significant SNP are reported.

**Table 2:** Independent SNPs associated with TSH and FT4 serum levels.

Gene	SNP	Chr	Position	A1/A2	Freq A1	Effect	StdErr	P	N	Het P
**TSH levels**										
*PDE8B*	rs6885099	5	76566105	A/G	0.594	−0.141	0.009	1.95×10^−56^	26042	0.520
*PDE10A*	rs753760	6	165966473	C/G	0.691	0.100	0.010	1.21×10^−24^	25988	0.363
*CAPZB*	rs10799824	1	19713761	A/G	0.161	−0.113	0.012	3.60×10^−21^	26031	0.042
*MAF/LOC440389*	rs3813582	16	78306854	T/C	0.674	0.082	0.010	8.45×10^−18^	25948	0.292
*VEGFA*	rs9472138	6	43919740	T/C	0.285	−0.079	0.010	6.72×10^−16^	25767	0.017
*VEGFA*	rs11755845	6	44012758	T/C	0.266	−0.065	0.010	1.68×10^−10^	25710	0.417
*NR3C2*	rs10032216	4	149888956	T/C	0.781	0.087	0.011	9.28×10^−16^	26053	0.504
*IGFBP5*	rs13015993	2	217333768	A/G	0.736	0.078	0.010	3.24×10^−15^	26016	0.605
*SOX9*	rs9915657	17	67639131	T/C	0.541	−0.064	0.009	7.53×10^−13^	25692	0.349
*NFIA*	rs334699	1	61393084	A/G	0.052	−0.141	0.021	5.40×10^−12^	25757	4.05×10^−3^
*FGF7*	rs10519227	15	47533656	A/T	0.245	−0.072	0.011	1.02×10^−11^	25988	0.098
*PRDM11*	rs17723470	11	45184143	T/C	0.279	−0.065	0.010	8.83×10^−11^	26054	0.833
*MIR1179*	rs17776563	15	86920108	A/G	0.322	−0.060	0.010	2.89×10^−10^	25758	0.452
*INSR*	rs4804416	19	7174848	T/G	0.569	−0.057	0.009	3.16×10^−10^	25632	0.438
*ABO*	rs657152	9	135129086	A/C	0.343	0.058	0.009	4.11×10^−10^	25765	1.22×10^−4^
*ITPK1*	rs11624776	14	92665344	A/C	0.660	−0.064	0.011	1.79×10^−9^	23482	0.845
*NRG1*	rs7825175	8	32535816	A/G	0.210	−0.066	0.011	2.94×10^−9^	25996	0.711
*MBIP*	rs1537424	14	35643769	T/C	0.608	−0.052	0.009	1.17×10^−8^	25478	0.333
*SASH1*	rs9497965	6	148562985	T/C	0.415	0.051	0.009	2.25×10^−8^	25980	0.444
*GLIS3*	rs1571583	9	4257209	A/G	0.249	0.057	0.010	2.55×10^−8^	25766	0.118
**FT4 levels**										
*DIO1*	rs2235544	1	54148158	A/C	0.510	0.138	0.012	7.87×10^−32^	17226	0.193
*LHX3*	rs7860634	9	138229500	A/G	0.530	0.102	0.013	2.30×10^−14^	14529	0.067
*FOXE1*	rs7045138	9	99631284	T/C	0.553	0.098	0.015	1.50×10^−11^	10997	0.457
*AADAT*	rs11726248	4	171290094	A/G	0.106	0.111	0.019	5.20×10^−9^	17515	0.972
*LPCAT2/CAPNS2*	rs6499766	16	54161629	A/T	0.478	0.056	0.012	1.18×10^−6^	17489	0.269
*NETO1/FBXO15*	rs7240777	18	69318732	A/G	0.5632	−0.049	0.012	3.13×10^−5^	17146	7.84×10^−3^

The table shows the association results for SNPs that reached genome-wide level (p<5×10^−08^) in the main meta-analysis. SNPs at *LPCAT2/CAPNS2* and *NETO1/FBXO15* reached the GW threshold in the gender-specific meta analysis (see Table 3), and here the p-value in the main meta-analysis is reported. For each SNP, the best candidate gene is showed, as well as its genomic position in build 36, the effect allele (A1) and the other allele (A2), its combined frequency across studies and its standard error, the effect size and its standard error, the p-value for association, the number of samples analyzed, and the p-values for heterogeneity of effects across the cohorts meta-analyzed. Effect sizes are standardized, so they represent the estimated phenotypic change, per each copy of the effect allele, in standard deviation units.

For TSH, 4 signals confirmed previously described loci with proxy SNPs at *PDE8B* (*P* = 1.95×10^−56^, r^2^ = 0.94 with the reported rs4704397), *CAPZB* (*P* = 3.60×10^−21^, r^2^ = 1 with the reported rs10917469) and *NR3C2* (*P* = 9.28×10^−16^, r^2^ = 0.90 with the reported rs10028213), whereas the signal was coincident at *MAF/LOC440389* (*P* = 8.45×10^−18^) [13]–[15]. The remaining signals were in or near 15 novel loci: *PDE10A (phosphodiesterase type 10A, P* = 1.21×10^−24^), *VEGFA* (*Vascular endothelial growth factor*, *P* = 6.72×10^−16^), *IGFBP5* (*insulin-like growth factor binding protein 5, P* = 3.24×10^−15^), *SOX9 (sex determining region Y-box 9*, *P* = 7.53×10^−13^), *NFIA* (*nuclear factor I*/*A*, *P* = 5.40×10^−12^), *FGF7* (*fibroblast growth factor 7*, *P* = 1.02×10^−11^), *PRDM11* (*PR domain containing 11*, *P* = 8.83×10^−11^), *MIR1179* (*microRNA 1179*, *P* = 2.89×10^−10^), *INSR* (*insulin receptor*, *P* = 3.16×10^−10^), *ABO (ABO glycosyltransferase*, *P* = 4.11×10^−10^), *ITPK1* (*inositol-tetrakisphosphate 1-kinase*, *P* = 1.79×10^−9^), *NRG1* (*neuregulin 1*, *P* = 2.94×10^−9^), *MBIP* (*MAP3K12 binding inhibitory protein 1*, *P* = 1.17×10^−8^), *SASH1* (*SAM and SH3 domain containing 1*, *P* = 2.25×10^−8^), *GLIS3* (*GLIS family zinc finger 3*, *P* = 2.55×10^−8^), (Figure 1, Figure 2, Figure 3, Figure 4).

For FT4, we confirmed the *DIO1* locus (*P* = 7.87×10^−32^), with the same marker previously reported in candidate gene studies [17], [18], and identified 3 additional novel loci, *LHX3* (*LIM homeobox 3*, *P* = 2.30×10^−14^), *FOXE1* (*forkhead box E1*, *P* = 1.50×10^−11^) and *AADAT* (*aminoadipate aminotransferase*, *P* = 5.20×10^−9^) (Figure 5). The most associated SNP at the *FOXE1* locus, rs7045138, is a surrogate for rs1443434(r^2^ = 0.97), previously only suggestively associated with FT4 levels [18], and is also correlated with SNPs recently reported to be associated with both low serum TSH and FT4 levels (r^2^ = 0.59 with rs965513) [19], as well as with hypothyroidism (r^2^ = 0.59 with rs7850258) [20].

At each locus, a single variant was sufficient to explain entirely the observed association, except for the *VEGFA* locus, which contained an independent signal located 150 kb downstream of the gene, detected by conditional analyses (Figure 1F and Table 2).

Of all 24 independent markers, significant evidence for heterogeneity (*P*<0.002, corresponding to a Bonferroni threshold of 0.5/24) was only observed at *ABO* (*P* = 1.22×10^−4^). Iodine nutrition, which may profoundly affect thyroid function, is quite different in some of the cohorts under study (i.e., Europe vs North America). To test whether the observed heterogeneity could be attributable to different iodine intake, we combined cohorts from South Europe (an iodine-deficient region) and compared effect sizes with those observed in a meta-analysis of North American samples (an iodine-replete region). Interestingly, the effect size of the top marker at *ABO* was three times larger in Europeans vs North American, and this difference remained significant after Bonferroni correction (*P* = 7.0.9×10^−4^) (Table S3). However, the relation of the *ABO* SNP, a tag for the blood group O, to iodine intake remains to be determined.

### Gender-specific analyses

**Figure 6:**
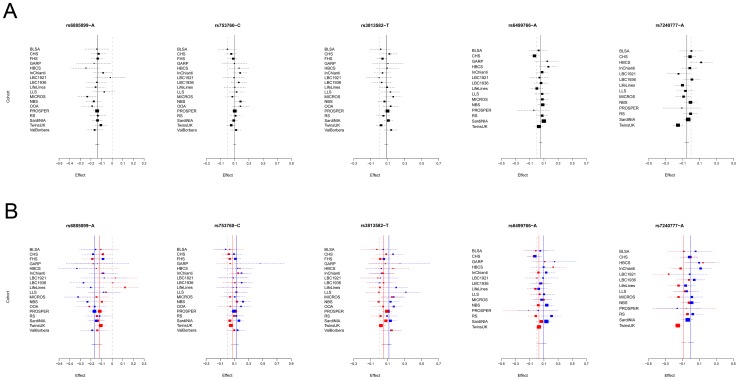
Forest plot of SNPs with gender-specific effects. Squares represent the estimated per-allele beta-estimate for individual studies (a) and in males and females separately (b). The area of the square is inversely proportional to the variance of the estimate. Diamonds represent the summary beta estimates for the subgroups indicated. Horizontal lines represent 95% confidence intervals. In b, red and blue dotted lines represent, respectively, females and males.

Given the reported clinical differences in thyroid function in males and females [21]–[23], we searched for gender-specific loci by whole-genome sex-specific meta-analysis, analyzing males and females separately in each cohort. Some of the loci detected in the main meta-analysis were seen at genome-wide significance level only in females (*NR3C2, VEGFA, NRG1* and *SASH1*) or in males (*MAF/LOC440389, FGF7*, *SOX9*, *IGFBP5*) with either the same top SNP or one surrogate, but effect sizes at their variants were significantly gender-specific only at *PDE8B*, *PDE10A* and *MAF/LOC440389,* considering a false discovery rate of 5% [24]. In addition, effects at *MAF/LOC440389* were significantly different also at the more stringent Bonferroni threshold of 1.9×10^−3^ ( = 0.05/26), and close to significance at *PDE8B* and *PDE10A* (Table 3). At these latter loci, the TSH-elevating alleles showed a stronger impact on trait variability in males compared to females (Figure 6). In addition, the gender specific meta-analysis for FT4, revealed a novel female-specific locus on chromosome 18q22, and a novel male-specific locus on chromosome 16q12.2, that had not been detected in the main meta-analysis (Table 3, Figure 6 and Figure S2). The female-specific signal (rs7240777, *P* = 3.49×10^−8^) maps in a “gene desert” region, with the nearest genes *NETO1* (*neuropilin (NRP) and tolloid (TLL)-like 1*), located, about 550 kb upstream and *FBXO15* (*F-box only protein 15*) 500 kb downstream (Figure 5D). The male-specific association is located in intron 11 of the *LPCAT2* (*lysophosphatidylcholine acyltransferase 2*) gene, and near *CAPNS2* (*calpain, small subunit 2*) (rs6499766, *P* = 4.63×10^−8^), a gene which may play a role in spermatogenesis [25]. The FT4-elevating alleles in the *NETO1/FBXO15* and *LPCAT2/CAPNS2* were fully gender-specific, i.e. there was no effect in males and in females, respectively (*P*>0.01).

**Table 3:** Top associated SNPs and their effect on males and females separately.

			Females				Males					
Gene	SNP	A1/A2	Effect	StdErr	P	N	Effect	StdErr	P	N	Het P	FDRs
**TSH levels**												
***PDE8B***	**rs6885099**	A/G	−0,120	0.012	6.09×10^−24^	14509	−0.168	0.013	2.70×10^−38^	11533	7.12×10^−3^	**0.037**
***PDE10A***	**rs753760**	C/G	0.076	0.013	4.64×10^−9^	14473	0.131	0.014	5.85×10^−20^	11515	5.40×10^−3^	**0.035**
*CAPZB*	rs10799824	A/G	−0.123	0.016	2.69×10^−14^	14504	−0.099	0.017	1.17×10^−8^	11527	0.309	0.618
***MAF/LOC440389***	**rs3813582**	T/C	0.055	0.013	1.75×10^−5^	14449	0.115	0.014	6.17×10^−17^	11499	**1.50×10^−3^**	**0.019**
*VEGFA*	rs9472138	T/C	−0.090	0.013	6.30×10^−12^	14291	−0.065	0.014	4.89×10^−6^	11476	0.208	0.450
*VEGFA*	rs11755845	T/C	−0.058	0.014	1.98×10^−5^	14250	−0.076	0.015	2.98×10^−7^	11460	0.368	0.683
*NR3C2*	rs10032216	T/C	0.106	0.014	1.72×10^−13^	14519	0.069	0.016	1.12×10^−5^	11534	0.092	0.294
*IGFBP5*	rs13015993	A/G	0.069	0.013	1.71×10^−7^	14491	0.095	0.015	7.60×10^−11^	11525	0.193	0.450
*SOX9*	rs9915657	T/C	−0.063	0.012	1.46×10^−7^	14241	−0.068	0.013	2.39×10^−7^	11451	0.793	0.896
*NFIA*	rs334699	A/G	−0.143	0.027	1.80×10^−7^	14253	−0.149	0.030	5.93×10^−7^	11504	0.874	0.909
*FGF7*	rs10519227	A/T	−0.051	0.014	3.80×10^−4^	14462	−0.095	0.015	6.09×10^−10^	11526	0.036	0.156
*PRDM11*	rs17723470	T/C	−0.069	0.013	2.92×10^−7^	14519	−0.056	0.015	1.45×10^−4^	11535	0.519	0.710
*MIR1179*	rs17776563	A/G	−0.053	0.013	3.70×10^−5^	14305	−0.069	0.014	6.16×10^−7^	11453	0.395	0.684
*INSR*	rs4804416	T/G	−0.058	0.012	1.76×10^−6^	14205	−0.058	0.013	1.12×10^−5^	11427	0.969	0.969
*ABO*	rs657152	A/C	0.054	0.013	1.31×10^−5^	14290	0.067	0.014	1.01×10^−6^	11475	0.498	0.710
*ITPK1*	rs11624776	A/C	−0.053	0.015	3.29×10^−4^	12255	−0.069	0.015	2.61×10^−6^	11227	0.453	0.693
*NRG1*	rs7825175	A/G	−0.084	0.015	1.64×10^−8^	14475	−0.049	0.016	2.36×10^−3^	11521	0.113	0.294
*MBIP*	rs1537424	T/C	−0.054	0.012	1.26×10^−5^	14091	−0.050	0.013	1.59×10^−4^	11387	0.848	0.909
*SASH1*	rs9497965	T/C	0.067	0.012	3.36×10^−8^	14462	0,031	0.013	0.023	11518	0.046	0.171
*GLIS3*	rs1571583	A/G	0.041	0.014	2.86×10^−3^	14290	0.074	0.015	9.82×10^−7^	11476	0.104	0.294
**FT4 levels**												
*DIO1*	rs2235544	A/C	0.130	0.015	2.62×10^−18^	10019	0.143	0.018	4.59×10^−15^	7201	0.605	0.786
*LHX3*	rs7860634	A/G	0.098	0.018	5.01×10^−8^	7665	0.108	0.019	1.72×10^−8^	6864	0.715	0.845
*FOXE1*	rs7045138	T/C	0.093	0.020	3.10×10^−6^	5801	0.105	0.021	4.96×10^−7^	5196	0.679	0.840
*AADAT*	rs11726248	A/G	0.123	0.024	4.03×10^−7^	10252	0.093	0.029	1.47×10^−3^	7263	0.440	0.693
***LPCT2/CAPNS2***	**rs6499766**	A/T	0.030	0.015	0.040	10231	0.099	0.018	4.63×10^−8^	7258	3.42×10^−3^	**0.029**
***NETO1/FBXO15***	**rs7240777**	A/G	−0.083	0.015	3.49×10^−8^	9963	−0.001	0.018	0.950	7183	**5.64×10^−4^**	**0.014**

The table shows the association results in males and females separately for all independent SNPs associated with TSH and FT4 in the main meta-analysis (Table 2), as well as for the marker found to be associated only in females in the gender-specific meta-analysis. The last two columns report the p-value (Het P) and the false discovery rates (FDRs) for differences of effect sizes. SNPs with significantly different effect sizes at 5% FDRs and/or Bonferroni threshold (p = 1.9×10^−3^) are highlighted in bold. SdtErr, standard error; A1, effect allele; A2 other allele.

Overall, the 20 TSH and the 6 FT4 associations account, respectively, for 5.64% and 2.30% of total trait variance.

### Common loci regulating TSH and FT4 levels

To explore overlap between TSH- and FT4-associated loci and their involvement in the HPT-negative feedback loop, we assessed the associations of the top TSH-associated SNPs on FT4 levels, and *vice versa*. For the SNPs in or near *PDE8B*, *MAF/LOC440389*, *VEGFA*, *IGFBP5*, *NFIA*, *MIR1179*, *MBIP* and *GLIS3* the TSH-elevating allele appeared to be associated with decreasing FT4 levels (*P*<0.05, Table S4). However, after application of Bonferroni correction (threshold for FT4 association of TSH SNPs, *P* = 2.5×10^−3^), none of these reciprocal associations remained significant.

By contrast, a positive relationship was seen for one of the FT4 associated loci, since the variant at the *LHX3* locus was significantly associated with higher levels of both FT4 and TSH (*P* = 5.25×10^−3^, with Bonferroni threshold 0.05/6 = 0.008).

As the presence of reciprocal associations between TSH and FT4 regulating SNPs would be expected from physiology, we tested the power of our study to detect such a relationship. Power calculation for the top SNP at *PDE8B*, which has the largest effect on TSH levels, revealed that our meta-analysis only has 9% power to detect an association of FT4 at a Bonferroni *P* = 2.5×10^−3^. We also carried out a bivariate analysis in the SardiNIA study using poly software to estimate specific contributions [26]. This analysis showed that most of the observed negative feedback correlation is due to environmental factors (environmental correlation = −0.130, genetic correlation = −0.065).

### Association of loci with hypothyroidism and hyperthyroidism

To assess possible clinical implications, we investigated whether the variants identified in individuals without overt thyroid pathologies (i.e., with TSH levels within the normal range and not taking thyroid medication) were also associated in individuals with abnormal TSH values (i.e., outside the reference range), who were not included in the initial meta-analysis as potentially affected by thyroid pathology. Towards this, we first assessed the global impact of TSH- and FT4-associated SNPs on the risk of increased or decreased TSH levels by comparing weighted genotype risk score (GRS) quartiles in the individuals with abnormal TSH values that were discarded for the GWAS analyses. For the TSH-associated SNPs, the odds of increased TSH levels were 6.65 times greater in individuals with a GRS in the top quartile compared to individuals in the bottom quartile (*P* = 3.43×10^−20^) (Table 4, top panel, lower vs upper tail). When we compared subjects with high TSH values with subjects within the normal TSH reference range, subjects with a GRS in the top quartile had odds of an elevated TSH 2.37 times greater than for subjects in the bottom quartile (*P* = 1.06×10^−17^) (Table 4). With regard to low TSH values versus the normal range, the odds ratio was 0.26 (*P* = 5.43×10^−13^) (Table 4, top panel, lower vs normal tail). By contrast, with the FT4-associated SNPs we found no significant associations for any of the tested comparisons (data not shown).

**Table 4:** TSH associated SNPs in extreme phenotype categories.

Quartile – based analysis	UPPER vs LOWER				UPPER vs NORMAL				LOWER vs NORMAL			
	OR	StdErr	P value	N (cases/controls)	OR	StdErr	P value	N (cases/controls)	OR	StdErr	P value	N (cases/controls)
Quartile1	–	–	–	141/169	–	–	–	215/2699	–	–	–	143/1842
Quartile2	2.16	0.17	7.09×10^−6^	194/108	1.31	0.10	0.010	280/2635	0.52	0.15	9.74×10^−6^	77/1913
Quartile3	3.20	0.18	1.51×10^−10^	219/86	1.43	0.11	7.00×10^−4^	311/2595	0.48	0.15	8.31×10^−7^	71/1913
Quartile4	6.65	0.21	3.43×10^−20^	256/49	2.37	0.10	1.06×10^−17^	447/2467	0.26	0.19	5.43×10^−13^	38/1947

Single marker analysis														
Gene	SNP	A1/A2	OR	StdErr	P value	N (cases/controls)	OR	StdErr	P value	N (cases/controls)	OR	StdErr	P value	N (cases/controls)
***PDE8B***	**rs6885099**	G/A	1.84	0.09	**7.33×10^−11^**	920/440	1.20	0.04	**3.61×10^−5^**	1363/11207	0.62	0.08	**2.85×10^−8^**	362/8426
***PDE10A***	**rs753760**	C/G	1.35	0.09	**1.74×10^−3^**	913/440	1.13	0.05	0.012	1356/11201	0.84	0.08	0.027	362/8420
***CAPZB***	**rs10799824**	G/A	1.78	0.11	**1.34×10^−7^**	915/440	1.27	0.06	**2.40×10^−4^**	1358/11185	0.68	0.09	**3.23×10^−5^**	362/8404
*MAF/LOC440389*	rs3813582	T/C	1.16	0.09	0.101	918/441	1.08	0.05	0.131	1361/11228	0.93	0.08	0.348	363/8447
***VEGFA***	**rs9472138**	C/T	1.29	0.09	5.21×10^−3^	919/441	1.11	0.05	0.042	1362/11228	0.78	0.08	**2.02×10^−3^**	363/8447
*VEGFA*	rs11755845	C/T	1.15	0.10	0.147	919/441	1.10	0.05	0.064	1362/11223	0.90	0.09	0.236	363/8442
***NR3C2***	**rs10032216**	T/C	1.27	0.10	0.022	920/441	1.26	0.06	**5.12×10^−5^**	1363/11229	0.91	0.09	0.290	363/8448
*IGFBP5*	rs13015993	A/G	1.19	0.10	0.086	920/440	1.12	0.05	0.020	1363/11224	0.92	0.09	0.317	362/8443
*SOX9*	rs9915657	C/T	1.15	0.09	0.105	911/440	1.04	0.04	0.326	1354/11211	0.91	0.08	0.193	362/8430
***NFIA***	**rs334699**	G/A	1.85	0.20	**2.10×10^−3^**	912/437	1.38	0.11	4.56×10^−3^	1355/11181	0.83	0.16	0.265	359/8400
***FGF7***	**rs10519227**	T/A	1.51	0.10	**3.09×10^−5^**	902/429	1.15	0.06	0.015	1345/11029	0.70	0.09	**4.84×10^−5^**	351/8248
*PRDM11*	rs17723470	C/T	1.21	0.10	0.056	920/441	0.97	0.05	0.558	1363/11228	0.78	0.09	3.33×10^−3^	363/8447
*MIR1179*	rs17776563	G/A	1.24	0.09	0.021	899/431	1.05	0.05	0.258	1342/11016	0.91	0.08	0.255	353/8235
*INSR*	rs4804416	G/T	1.14	0.09	0.123	920/441	1.10	0.04	0.034	1363/11229	0.97	0.08	0.677	363/8448
*ABO*	rs657152	A/C	1.12	0.10	0.247	919/441	1.04	0.04	0.358	1362/11219	0.90	0.09	0.228	363/8438
***ITPK1***	**rs11624776**	C/A	1.36	0.10	**2.44×10^−3^**	907/439	1.13	0.05	0.015	1350/11150	0.82	0.09	0.028	361/8369
*NRG1*	rs7825175	G/A	1.24	0.11	0.051	904/440	1.20	0.06	3.85×10^−3^	1347/11151	0.89	0.10	0.223	362/8370
*MBIP*	rs1537424	C/T	1.26	0.09	0.012	920/441	1.12	0.04	0.012	1363/11229	0.87	0.08	0.082	363/8448
*SASH1*	rs9497965	T/C	1.08	0.09	0.401	907/441	1.02	0.04	0.623	1350/8129	0.98	0.08	0.823	363/5348
*GLIS3*	rs1571583	A/G	0.97	0.10	0.769	916/438	1.06	0.05	0.273	1359/11206	1.01	0.09	0.891	360/8425

The table shows results for the quartile-based GRS scores (top panel) and single marker (bottom panel) analyses in extreme phenotype categories, defined as TSH >4 mIU/L (UPPER) or TSH <0.4 mIU/L (LOWER). NORMAL, individuals with TSH within the normal range. OR, odds ratio; StdErr, standard error. A1, effect allele; A2 other allele. SNPs reaching the Bonferroni significance threshold are highlighted in bold.

We also assessed the 20 independent TSH SNPs individually in relation to the risk of abnormal TSH levels by case-control meta-analysis in subjects with high (cases) versus low (controls) TSH values. This analysis showed that variants at *PDE8B*, *CAPZB*, *FGF7*, *PDE10A, NFIA* and *ITPK1* loci are significantly associated (Bonferroni threshold *P* = 2.5×10^−3^) with abnormal TSH levels (Table 4, bottom panel). *PDE8B*, *CAPZB* and *FGF7* were also strongly associated with the risk of decreased TSH levels in an analysis of individuals with low (cases) versus normal range TSH (controls). In addition, variants at *VEGFA* were also significantly associated in this comparison. Finally, when individuals with high TSH values were analyzed versus controls, the *NR3C2* locus appeared significantly associated in addition to *PDE8B* and *CAPZB*.

### Association of TSH lead SNPs in pregnant women

Normal thyroid function is particularly important during pregnancy and elevated TSH levels are implicated in a number of adverse outcomes for both mother and offspring. We therefore assessed whether the TSH lead SNPs were also associated with elevated TSH during pregnancy, when increased TH production is necessary. We tested 9 of the 20 lead TSH variants (or their proxies, see Text S1) in a cohort of 974 healthy pregnant women at 28 weeks gestation [27] and found, as expected, that mean TSH levels were correlated with the number of TSH-elevating alleles (*P* = 3.0×10^−12^, Table S5). Effect size estimates in pregnant women were not significantly different when compared to those of women in the main gender-specific meta-analysis (heterogeneity *P* value>0.05), suggesting that the effects of the TSH-elevating alleles are no greater during pregnancy (data not shown). However, there was evidence of association between the number of TSH-raising alleles and subclinical hypothyroidism in pregnancy, both in the whole sample (OR per weighted allele: 1.18 [95%CI: 1.01, 1.37], *P* = 0.04) and in TPO antibody-negative women (1.29 [95%CI: 1.08, 1.55], *P* = 0.006) (Table S6).

## Discussion

We report 26 independent SNPs associated with thyroid function tests in euthyroid subjects, 21 of which represent novel signals (16 for TSH and 5 for FT4). Overall they explain 5.64% and 2.30% of the variation in TSH and FT4 levels, respectively.

We observed that carriers of multiple TSH-elevating alleles have increased risk of abnormal TSH levels, and also found association between the number of TSH-elevating alleles and subclinical hypothyroidism in pregnancy. These results are potentially clinically relevant, because abnormal TSH values are the most sensitive diagnostic markers for both overt and subclinical thyroid disease [4]. The variants identified in the current study, or those in LD with them, may thus contribute to the pathogenesis of thyroid disease. Of note, we found eight loci significantly associated with abnormal TSH levels (*PDE8B*, *PDE10, CAPZB, VEGFA*, *NR3C2, FGF7*, *NFIA* and *ITPK1*), of which two were specifically associated with either abnormally low (*VEGFA*) or elevated (*NR3C2*) TSH values, suggesting differential mechanisms for the contribution of these variants to hyper- and hypothyroidism, respectively. Interestingly, the mineralocorticoid receptor *NR3C2* gene has recently been found to be up-regulated in adult-onset hypothyroidism [28], and *PDE8B* and *CAPZB* have been suggestively associated with hypothyroidism by GWAS [29]. Alternatively, it may be that carriers of these alleles are healthy individuals who may be misdiagnosed as having thyroid disease because their genetically determined TSH concentrations fall outside the population-based reference range. More research is required to determine which of these interpretations is correct, and the relevance of these variants as markers for thyroid dysfunction or thyroid-related clinical endpoints.

The evidence for gender-specific differences at several TSH and FT4 regulatory loci is intriguing. They included variants at *PDE8B*, *PDE10A*, and *MAF/LOC440389*, which showed significantly stronger genetic effects with pituitary-thyroid function in males, and variants at *NETO1/FBX015* and *LPCAT2/CAPNS2* which seems to have an effect only in females and males, respectively. Sex differences in the regulation of thyroid function have generally been linked to the influence of sex hormones and autoimmune thyroid disease, resulting in a higher prevalence of thyroid dysfunction in women, without clear understanding of underlying molecular mechanisms [21]–[23]. Our study suggests that differential genes and mechanisms are potentially implicated in the regulation of thyroid function in men and women. Given the impact of thyroid function on several disease outcomes as well as male and female fertility and reproduction, clarifying the underlying associations may provide additional insight for future interventions.

Although it is well known that TSH and FT4 levels are tightly regulated through a negative feedback loop involving the HPT axis, we detected significant overlap between TSH and FT4 signals only at the *LHX3* locus, which was primarily associated in our study with FT4. The *LHX3* allele is associated with an increase of both TSH and FT4, which is consistent with the essential role of this transcription factor in pituitary development. Inactivating mutations in *LHX3* cause the combined pituitary hormone deficiency-3 syndrome [CPHD3 (MIM#221750)] [30], [31], characterized by low TSH and FT4 levels. The positive association of the *LHX3* variant with both TSH and FT4 suggests an effect of this allele at the level of the HPT-axis, resulting in an increased exposure to thyroid hormone throughout life. In contrast, although several of the TSH-elevating alleles appeared to be associated with decreasing FT4 levels, none of these reciprocal associations remained significant after Bonferroni correction. Lack of loci associated in a reciprocal manner with both TSH and FT4 is somewhat puzzling, as their presence would be expected from physiology. However, these findings are consistent with initial reports by Shields et al. [27] and more recent findings by Gudmundsson et al. [32]. A power analysis showed that our study – in spite of being one of the larger conducted so far on these traits – is underpowered to detect an inverse relationship between TSH and FT4 variants, considering a Spearman rank correlation of −0.130 between these traits [12]. As a consequence, contrasting studies on smaller sample sizes may also lack power and cannot be considered robust when testing this relationship [33]. In addition, we estimated that most of the observed negative feedback correlation is due to environmental factors; so it is unlikely that negative feedback is controlled by a genetic locus with large effect. This observation can rationalize the lack of reciprocal, significant associations detected for both TSH and FT4 in this and other studies, and further supports the crucial role of the HPT-axis in maintaining normal levels of thyroid hormone.

At present the relationship between the associated variants and specific mechanisms involved in regulating TSH and FT4 levels has not been established, but we have identified strong candidates at the majority of the loci by literature-mining approaches, as detailed below and in Table 5.

**Table 5:** Candidate genes at newly discovered loci for TSH and FT4 levels.

SNP	Region	Gene	Position	Trait	Function
rs753760	6q26	*PDE10A*	intron 1	TSH	Encodes a dual specificity phosphodiesterase abundant in the thyroid, which can hydrolyze both cAMP and cGMP to the corresponding nucleoside 5′ monophosphate, but has higher affinity for cAMP, and is more efficient with cAMP as substrate. This gene was previously suggestively associated with TSH levels and hypothyroidism and linkage has been observed over this gene in families with individuals reaching the clinical criteria for sub-clinical and clinical thyroid disorders [13], [34]. The top SNP is only weakly correlated with previously reported variants (r2 = 0.449 with rs2983521 and r2 = 0.184 with rs9347083), but is a perfect proxy of a SNP recently reported in association with TSH [32].
rs9472138; rs11755845	6p12	*VEGFA*	intergenic	TSH/FT4	Encodes a growth factor implicated in angiogenesis, which acts as an important regulator of both benign and malignant processes in the thyroid [62]. Angiogenesis is particularly critical for thyroid function, as the local microvasculature exerts an essential role in the continuous supply of iodine, the key element of thyroid hormone synthesis. In response to a reduction in intracellular iodine concentration, thyrocytes rapidly release angiogenic signals, including an increase in *VEGFA* expression and secretion [63], [64]. Notably, thyroid hormone stimulation in rat brain has been shown to induce *VEGFA* upregulation [65], which is consistent with the nominal association of the *VEGFA* locus with FT4 levels observed in this study. The two independent signals, rs9472138 and rs11755845, associated with TSH levels map 40 kb downstream of *VEGFA*, which is the nearest gene in the region. SNPs in this region were recently reported in association with TSH levels (r2 = 0.874 and r2 = 0.947) [32].
rs13015993	2q33-36	*IGFBP5*	intergenic	TSH	It belongs to a protein family that interacts with insulin-like growth factors (IGFs) and plays a major role in regulating cell proliferation, differentiation, apoptosis and transformation. *IGFBP5* is significantly over expressed in thyroid papillary carcinoma [54], [66]. Studies *in vitro* showed that TSH, through cAMP, inhibits *IGFBP5* transcription, whereas enhanced production of IGFBP5 is correlated with inhibition of thyroid function [67];[68]. In addition, *IFGBP5* has been found up-regulated in response to thyroid hormone in bone, where it interacts with the growth hormone/insulin-like growth factor (GH/IGF) system to contribute to bone formation [69], suggesting that thyroid hormone may potentate the effect of IGF-1 at the receptor level. The top SNP maps about 60 kb upstream of *IGFBP5*. A proxy of this SNP has been recently found associated with TSH and FT4 levels (rs737308, r2 = 0.927) [32].
rs9915657	17q23	*SOX9*	3′UTR	TSH	Encodes a transcription factor involved in chondrocyte differentiation and male sex determination, although other specific functions are known. The TA domain of SOX9, which is expressed both in the pituitary and in the thyroid, has been reported to interact with a component of the thyroid hormone receptor complex (TRAP230) [70]. How this interaction could affect TSH levels is at present unclear. The top SNP maps 5 kb downstream of *SOX9*, which is the nearest gene in the region.
rs334699	1p31.3-p31.2	*NFIA*	intron 3	TSH	Encodes a member of the NF1 (nuclear factor 1) family of transcription factors. NFI proteins have been implicated in regulating developmental processes by their specific expression pattern during embryonic development and by analysis of NFI-deficient mice [71]. In addition, they play crucial roles in the transcription of many cellular genes. Members of this family, including NFIA, have been shown to interact with thyroid transcription factor 1 (TTF1) [72], a transcription factor essential for thyroid-specific gene expression [73]. The top SNP maps in intron 3 of the gene. A proxy of this SNP has been recently found associated with TSH levels (rs334725, r2 = 1) [32].
rs10519227	15q21.2	*FGF7*	intron 2	TSH	Encodes a member of the fibroblast growth factor (FGF) family. FGF family members are involved in a variety of biological processes, including embryonic development, cell growth, morphogenesis, tissue repair, tumor growth and invasion. FGF signals play a role in the development of the thyroid gland and mice deficient for corresponding receptors show thyroid agenesis [74]. The top SNP maps in intron 2 of *FGF7*. A proxy of this SNP has been recently associated by GWAS with thyroid volume and goiter (rs4338740, r2 = 0.874) [36].
rs17723470	11p11	*PRDM11*	intron 2	TSH	Encodes a member of the family of PR-domain genes involved in human cancers [75]. The function of *PRDM11* and its correlation with TSH levels is unclear; however the association with low TSH values in the extreme phenotype analysis supports a role of this gene in this trait. The top SNP maps in intron 2 of the gene and is moderately correlated with a SNP associated with TSH levels (rs7128207, r2 = 0.55) [32].
rs17776563	15q25.3	*MIR1179*	intergenic	TSH	Encodes a microRNAs (miRNAs), which are short non-coding RNAs involved in post-transcriptional regulation of gene expression in multicellular organisms by affecting both the stability and translation of mRNAs. The associated SNP maps about 30 kb upstream of the gene, which is the nearest gene in the region.
rs4804416	19p13.3-13.2	*INSR*	intron 2	TSH	Encodes the insulin receptor precursor, which is post-translationally cleaved after removal of the precursor signal peptide into two chains (alpha and beta) that are covalently linked. Binding of insulin to the insulin receptor (INSR) stimulates glucose uptake. Two transcript variants encoding different isoforms have been found for this gene. INSR isoforms appear overexpressed in thyroid tumors, where they interact with insulin homolog IGFs (I and II), which act as potent mitogenic and antiapoptotic factors in a variety of human malignancies, and supporting a specific role of the GH/IGF pathway in thyroid function. The top SNP maps in intron 2 of the INSR, and is moderately correlated (rs10420008, r2 = 0.435) with a variant recently associated with TSH levels [32].
rs657152	9q34.2	*ABO*	intron 1	TSH	Encodes proteins related to the blood group system, ABO, which determines the individual blood group. The associated SNP is in intron 1 of the gene and is a tag of the O blood group allele, caused by a deletion of guanine-258 near the N-terminus of the protein which results in a frameshift and translation of an almost entirely different protein. The correlation of TSH with ABO blood groups is currently unclear. *ABO* has been found associated with several phenotypes, including serum levels of different molecules [76]. It was reported that serum levels of TSH vary in individuals with ABO blood types, and that blood group O may be associated with hyperthyroidism [77].
rs11624776	14q31	*ITPK1*	intergenic	TSH	Encodes the enzyme inositol 1,3,4-trisphosphate 5/6-kinase, which catalyzes the rate-limiting step in the formation of higher phosphorylated forms of inositol in mammalian cells. ITPK1 plays a pivotal role in inositol metabolism and mice producing reduced levels of ITPK1 develop neural tube defects [78]. Its role in the regulation of TSH levels is at present unclear. Of note, inositol phosphates/Ca2+ cascades mediates TSH action on thyroid hormone synthesis [79] The associated SNP is located about 15 kb upstream of the gene, which is the nearest gene in the region.
rs7825175	8p12	*NRG1*	intron 2	TSH	Encodes neoregulin 1, a glycoprotein that interacts with the NEU/ERBB2 receptor tyrosine kinase to increase its phosphorylation on tyrosine residues. *NRG1* is a signaling protein that mediates cell-cell interactions and plays critical roles in the growth and development of multiple organ systems. Its gene dysregulation has been linked to cancer, schizophrenia and bipolar disorder. The associated SNP maps in intron 2 of the gene and is only weakly correlated with a variant recently associated with TSH levels and thyroid cancer risk (rs2439302, r2 = 0.257) [32].
rs1537424	14q13.3	*MBIP*	intergenic	TSH	Encodes MAP3K12/MUK-binding inhibitory protein, a MAP3K regulator during osmolarity sensing and stress signaling that localizes in either the cytoplasm or nucleus [80]. SNPs in this locus have been recently reported as associated with TSH levels and thyroid cancer risk [32]. The top associated SNP maps at about 190 kb downstream of *MBIP*, which is the nearest gene in the region. Recently, a long, intergenic, noncoding RNA gene (lincRNA) named *Papillary Thyroid Carcinoma Susceptibility Candidate 3* (*PTCSC3*) has been mapped 3.2 kb downstream of rs944289 (r2 = 0.708 with our top SNP), whose expression is strictly thyroid specific and acts as a PTC tumor suppressor gene [81].
rs9497965	6q24.3	*SASH1*	intergenic	TSH	Encodes a member of the SLY-family of signal adapter proteins and is a candidate tumor suppressor in breast and colon cancer. However, the biological function of SASH1 and its involvement in malignant transformation remain largely unknown. Of note, SASH1 has been identified as a downstream target of the insulin/IGF1/PI 3-kinase signaling pathway [82], which appears implicated in TSH levels in the current study. The associated SNP is located about 130 kb upstream of *SASH1*, which is the nearest gene in the region.
rs1571583	9p24.2	*GLIS3*	intron 2	TSH	Encodes a nuclear protein with five C2H2-type zinc finger domains, which is a member of the GLI-similar zinc finger protein family. GLIS3 functions as both a repressor and activator of transcription and is specifically involved in the development of pancreatic beta cells, the thyroid, eye, liver and kidney. Mutations in this gene have been associated with neonatal diabetes and congenital hypothyroidism (NDH) [52]. The top SNP at this locus maps in intron 2 of the gene.
rs7860634	9q34.3	*LHX3*	intron 6	FT4/TSH	Encodes a transcription factor with an essential role in pituitary development [30]. Mutations in *LHX3* cause the combined pituitary hormone deficiency-3 (CPHD3) syndrome (OMIM #221750), characterized by a complete deficit in growth hormone, prolactin, gonadotropin, and TSH, a rigid cervical spine leading to limited head rotation, as well as an extended spectrum with variable sensorineural hearing loss and ACTH deficiency [31], [42], [43], which is consistent with its association also with TSH levels observed in this study. This locus has been also recently associated with height in Japanese [44] The top SNP maps in intron 6 of the gene.
rs7045138	9q22	*FOXE1*	intergenic	FT4	Encodes a transcription factor crucial for thyroid morphogenesis. Mutations in *FOXE1* cause the Bamforth-Lazarus syndrome (MIM #241850), characterized by thyroid dysgenesis with neonatal hypothyroidism, cleft palate, choanal atresia and spiky hair [40], [41]. In addition, *FOXE1* is a susceptibility locus for thyroid cancer [19], [83]–[85]. The top SNP maps 25 kb upstream of *FOXE1* and is also associated in our study with FT4 levels. SNPs in the *FOXE1* locus were previously associated with FT4 levels in a recent candidate gene analysis (rs1443434, r2 = 0.776) [18], as well as by GWAS with both low serum TSH and T4 levels (rs965513, r2 = 1) [19], and hypothyroidism (rs7850258, r2 = 0.625) [20].
rs716822	4q33	*AADAT*	intron 4	FT4	AADAT catalyzes the synthesis from kynurenine (KYN) of kynurenic acid (KYNA), which is implicated in the pathophysiology of several diseases of the central nervous system involving inflammation-induced brain injury 46–49. The KYN pathway has been associated with the induction of proinflammatory cytokines in the brain, which are known to activate the hypothalamo-pituitary-adrenal (HPA)-axis, involved in stress response and affecting the HPT-axis and thyroid function, including FT4 levels [45]. The top SNP maps in intron 4 of the gene.
rs7240777	18q22	*NETO1/FBXO15*	intergenic	FT4	The top SNP maps in a gene desert region, with *NETO1* located about 550 kb upstream and *FBXO15* about 500 kb downstream. None of these genes has a clear role in thyroid function. *NETO1* is expressed in brain and encodes a predicted transmembrane protein containing two extracellular CUB domains followed by a low-density lipoprotein class A (LDLa) domain. A similar gene in mice plays a critical role in spatial learning and memory by regulating the function of synaptic N-methyl-D-aspartic acid receptor complexes in the hippocampus. *FBOX15* encodes a member of the F-box protein family characterized by an approximately 40-amino acid F-box motif. SCF complexes, formed by SKP1 (MIM#601434), cullin (see CUL1; MIM#603134), and F-box proteins, act as protein-ubiquitin ligases. F-box proteins interact with SKP1 through the F box, and they interact with ubiquitination targets through other protein interaction domains.
rs6499766	16q12.2	*LPCAT2/CAPNS2*	intron 11	FT4	*LPCAT2* encodes a member of the lysophospholipid acyltransferase family. The enzyme may function to catalyze both the biosynthesis of platelet-activating factor and of glycerophospholipid precursors from arachidonyl-CoA and lysophosphatidylcholine. The encoded protein may function in membrane biogenesis and production of platelet-activating factor in inflammatory cells. The associated SNP maps in intron 11 of the gene, and is also near *CAPNS2*, which is contained in the *LPCAT2* gene. None of these genes has a clear role in thyroid function. Of note, calpain subunit genes (*CAPNS1* and *CAPNS2*) play important functions in mammalian reproduction [25].

The table lists genes of interest in the novel associated regions. For each associated region, the reported gene either contains the lead SNP or is in closest physical proximity with the lead SNP.

Most of the 16 novel loci implicated in the regulation of TSH are highly represented in the thyroid with the exception of *PRDM11*, expressed in brain, *ABO*, in blood, and *MIR1179*. *PDE10A* encodes a cAMP-stimulated phosphodiesterase, which was previously only suggestively associated with TSH levels and hypothyroidism [13], [34], although the tested variants were weakly correlated with our top signal (r^2^ = 0.55 with rs2983521 and r^2^ = 0.15 with rs9347083). The presence of linkage at this gene in families reaching accepted clinical criteria of thyroid dysfunction reinforces the observation that variants in this gene may contribute to clinical thyroid disorders [34]. *PDE10A*, together with *PDE8B* and *CAPZB*, emerged in our study as the strongest currently known genetic determinants of this trait. Both *PDE8B* and *PDE10A* are implicated in cAMP degradation in response to TSH stimulation of thyrocytes. In addition, the activity of both *PDE10A* and *CAPZB* appear modulated by cAMP [35], [36]. These three genes most likely act in a pathway that leads to cAMP-dependent thyroid hormone synthesis and release, thus highlighting a critical role of cAMP levels in thyroid function. For the other TSH-associated loci (*VEGFA*, *IGFBP5*, *SOX9, NFIA, FGF7, PRDM11, MIR1179, INSR*, *ABO*, *ITPK1*, *NRG1*, *MBIP*, *SASH1* and *GLIS3*), hypotheses can be formulated based on the published literature (see Table 5), but further studies will be necessary to clarify the exact biological mechanisms and the specific genes involved at each locus. The association of TSH levels with *IGFBP5*, *INSR* and *NR3C2* is, however, an indication of a specific role of the growth hormone/insulin-like growth factor (GH/IGF) pathway in thyroid function. Remarkably, expression of *IGFBP5* is tightly regulated by cAMP, again underlying the pivotal role of this second messenger in determining net TSH levels [37].

For FT4, the *DIO1*, *FOXE1* and *LHX3* identified loci have strong biological support as potential effectors. While both *DIO1* and *FOXE1* were previously associated with FT4 levels and hypothyroidism by candidate gene analysis and functional studies [17]–[19], [38]–[41], association at *LHX3* is novel and is consistent with the essential role of this transcription factor in pituitary development (see above) [30], [31], [42], [43]. Consistent with the role of pituitary in growth, this locus has also recently been associated with height in Japanese [44]. The associations of *AADAT*, *NETO1*/*FXBO15* and *LPCT2/CAPNS2* with FT4 levels are currently less clear. It may be relevant that AADAT catalyzes the synthesis of kynurenic acid (KYNA) from kynurenine (KYN), a pathway that has been associated with the induction in brain of proinflammatory cytokines that are known to activate the hypothalamo-pituitary-adrenal (HPA) axis, in turn affecting the HPT axis and thyroid function, including FT4 levels [45]–[49].

Additional pathway analyses by MAGENTA [50], GRAIL [51], and IPA (Ingenuity Systems, www.ingenuity.com) to look for functional enrichment of the genes mapping to the regions associated with TSH, FT4 or both, yielded no novel interactions. However, IPA highlighted an over-representation of genes implicated in developmental processes (11/26, *P* = 6.27×10^−6^–8.85×10^−3^) and cancer (16/26 loci, *P* = 2.44×10^−6^–9.30×10^−3^). This is consistent with the notion that a normally developed thyroid gland is essential for both proper function and thyroid hormone synthesis, and that defects in any of the essential steps in thyroid development or thyroid hormone synthesis may result in morphologic abnormalities, impaired hormonogenesis and growth dysregulation. It is also interesting to note that 11 of the 20 TSH signals and 3 of the 6 FT4 signals are connected in a single protein network, underlying the biological interrelationship between genes regulating these traits (Figure S3).

While our manuscript was in preparation, a GWAS of comparable sample size was published on levels of TSH in the general Icelandic population, which confirmed 15 of our reported loci (E. Porcu et al., 2011, ESHG, abstract), and inferred a role for three TSH-lowering variants in thyroid cancer [32]. Four additional TSH loci identified by Gudmundsson and colleagues were also associated in our sample-set of euthyroid individuals with p<0.05 and consistent direction of effects (*VAV3*, *NKX2–3*, *TPO* and *FOXA2*). Finally, 2 loci (*SIVA1*, *ELK3*) could not be tested because the corresponding SNPs or any surrogate (r2>0.5) were not available in our data set (Table S7). Our study shows that most of the loci described in Icelanders are reproducible in other populations of European origin; differences in sample size, phenotype definition (i.e., selection of euthyroid subjects vs general population) and in the genetic map used to detect associations most likely explain non-overlapping genome-wide significant signals. Among them, the reported signals at *SOX9*, *ABO*, *SASH1*, *GLIS3* and *MIR1179* will need to be confirmed in other studies; but one of them - *GLIS3*- is a prime candidate, because it is involved in congenital hypothyroidism [52]. Interestingly, despite the use of variants detected through whole-genome sequencing in Icelanders, the top signals at seven overlapping loci (*PDE8B*, *PDE10A*, *CAPZB*, *MAF*/*LOC440389*, *VEGFA*, *NR3C2*, *IGFBP5*) were either coincident or in high LD (r2>0.9) with those detected in our HapMap-based meta-analysis. Thus, such variants are likely to be the causative ones.

In conclusion, our study reports the first GWAS meta-analysis ever carried out on FT4 levels, adds to the existing knowledge novel TSH- and FT4-associated loci and reveals genetic factors that differentially affect thyroid function in males and females. Several detected loci have potential clinical relevance and have been previously implicated both in Mendelian endocrine disorders (*LHX3* [MIMM#221750], *FOXE1* [MIMM#241850], *PDE8B* [MIMM#614190], *NR3C2* [MIMM#177735], *INSR* [MIMM#609968], *GLIS3* [MIMM#610199]) and thyroid cancer (*FOXE1* [19], *VEGFA* [53], *IGFBP5* [54], *INSR* [55], *NGR1* [32], *MBIP* [32], *FGF7* [56]). Furthermore, the TSH-associated variants were found to contribute to TSH levels outside the reference range. Overall, our findings add to the developing landscape of the regulation of hypothalamic-pituitary-thyroid axis function and the consequences of genetic variation for hypo- or hyperthyroidism.

## Methods

### Ethics statement

All human research was approved by the relevant institutional review boards, and conducted according to the Declaration of Helsinki.

### Cohort details

Cohort description, genotyping and statistical methods for individual study cohorts are reported in Text S1 and Table S1.

### Statistical analyses

We carried out a meta-analysis including up to 26,523, individuals from 18 cohorts for TSH and up to 17,520 individuals from 15 cohorts for FT4 (see Table 1). FT4 measures were not available for all 21,955 individuals with TSH levels of the 15 participating cohorts. We combined evidence of associations from single GWAS using an inverse variance meta-analysis, where weights are proportional to the squared standard error of the beta estimates, as implemented in METAL [57]. Prior to GWAS, each study excluded individuals with known thyroid pathologies, taking thyroid medication, who underwent thyroid surgery, and with out-of-range TSH values (<0.4 mIU/L and >4 mIU/L), and an inverse normal transformation was applied to each trait (Table S1). Age, age-squared, and gender were fitted as covariates, as well as principal components axes or additional variables, as required (Table S1). Family-based correction was applied if necessary (see Table S1). Uniform quality control filters were applied before meta-analysis, including MAF <0.01, call rate <0.9, HWE *P*<1×10^−6^ for genotyped SNPs and low imputation quality (defined as r^2^<0.3 or info <0.4 if MACH [58] or IMPUTE [59], [60] were used, respectively) for imputed SNPs.

Genomic control was applied to individual studies if lambda was >1.0. The overall meta-analysis showed no significant evidence for inflated statistics (lambda for TSH, FT4 and were 1.05 and 1.03 respectively). To evaluate for heterogeneity in effect sizes across populations, we used a chi-square test for heterogeneity, implemented in METAL [57]. The same test was used to evalute heterogeneity related to iodine intake, by comparing effect sizes obtained in a meta-analysis of studies assessing individuals from South Europe (InChianti, MICROS, Val Borbera, SardiNIA, totaling up to 7,488 subjects) with those estimated in a meta-analysis of studies assessing individuals from North America (BLSA, CHS, FHS, OOA, totaling up to 5,407 subjects). Finally, the main meta-analysis was carried out independently by two analysts who obtained identical results.

### Conditional analysis

To identify independent signals, each study performed GWA analyses for both TSH and FT4 by adding the lead SNPs found in the primary analysis (19 for TSH, and 4 for FT4, see Table 2) as additional covariates to the basic model, and removing those from the test data set. When lead SNPs were not available, the best proxies (r^2^>0.8) were included. We then performed a meta-analysis on the conditional GWAS results, using the same method and filters as described above. We used the standard genome-wide significance cutoff (*P*<5×10^−8^) to declare a significant secondary association.

### Gender-specific analysis

To identify sex-specific effects, each study performed GWA analyses for each gender separately, using the same covariates and transformation as in the basic model (with the exception of gender covariate). We then performed a meta-analysis on association results using the same method and filters described for the primary analysis. To evaluate sex-specific differences we tested heterogeneity between effect sizes as described above. False-discovery rates (FDRs) on the 26 associated SNPs were calculated with R's p.adjust() procedure via the method of Benjamini and Hochberg [24].

### Variance explained

The variance explained by the strongest associated SNPs was calculated, for each trait and in each cohort, as the difference of R^2^ adjusted observed in the full and the basic models, where the full model contains all the independent SNPs in addition to the covariates. The estimates from each cohort were combined using a weighted average, with weights proportional to the cohort sample size.

### Extreme phenotype analysis

To evaluate the impact of the detected variants with clinically relevant TSH levels, we compared the allele frequencies observed in different categories of individuals in a case-control approach. Specifically, we compared individuals in the upper and lower TSH tails (individuals with TSH >4 mIU/L and TSH <0.4 mIU/L, respectively, whom were excluded for the GWAS analyses), as well as individuals in each tail with those in the normal TSH range. In the first case, individuals in the lower tail were considered controls and those in the upper tail cases. In the other two cases, we defined individuals in the normal range as controls and individuals on the two tails cases. To avoid sources of bias, individuals taking thyroid medication and/or with thyroid surgery were excluded. Only unrelated individuals were selected from the family-based cohort SardiNIA, while GEE correction was applied to the TwinsUK dataset. Results from single cohorts were then meta-analyzed. We first assessed the global impact of the 20 TSH- and 6 FT4-associated variants by defining a genotype-risk score (GRS) for each individual as the weighted sum of TSH- and FT4-elevating alleles, with weights proportional to the effect estimated in the meta-analysis. For each comparison, we then calculated quartiles from the global distribution (cases+controls) of the genotype score and used quartile 1 as the baseline reference to compare the number of cases and controls in the other quartiles. In addition, for TSH-associated variants we conducted single SNP comparisons. GRS quartile and single SNP analyses were performed by each study separately. Cohort specific results were then meta-analyzed for both the GRS score and single SNP results only if they had at least 50 cases and 50 controls. Specifically, cohorts included were: CHS, Lifelines, PROSPER, RS, SardiNIA and TwinsUK.

### Bivariate analysis

Bivariate analysis was carried out with the software poly [26] in the SardiNIA cohort using the same individuals included in the GWAS and considering the same covariates and transformation for TSH and FT4 levels.

### Web resources

The URLs for data presented herein are as follows:

METAL, http://www.sph.umich.edu/cgs/abecasis/metal

MACH, http://www.sph.umich.edu/csg/abecasis/MACH/

IMPUTE, https://mathgen.stats.ox.ac.uk/impute/impute.html

LocusZoom, http://csg.sph.umich.edu/locuszoom/

HapMap, http://www.hapmap.org

Online Mendelian Inheritance in Man (OMIM), http://www.omim.org/

## Supporting Information

Figure S1 Manhattan plots from meta-analysis results of serum TSH (panel A) and FT4 (panel B) levels. SNPs are plotted on the x axis according to their position (build 36) on each chromosome against association with TSH (A) and FT4(B) on the y axis (shown as –log10 P value) in. The loci highlighted in green are those that reached genome-wide significance (P<5×10^−8^). In each panel, quantile-quantile plots obtained with all SNPs (red dots) and after removal of SNPs within associated regions (blue dots) are also shown. The gray area corresponds to the 90% confidence region from a null distribution of P values (generated from 100 simulations).
(TIF)

Figure S2 Panel A. Manhattan plots from meta-analysis results of serum TSH and FT4 levels for men and women separately are shown as indicated. SNPs are plotted on the x axis according to their position (build 36) on each chromosome; association with TSH and FT4 is indicated on the y axis (as –log10 P value). Signals reaching genome-wide statistical significance in the gender specific analysis are shown in green. Panel B. Quantile-quantile plots are shown for all SNPs (red dots) and after removal of SNPs within associated regions (blue dots).
(TIF)

Figure S3 Ingenuity pathway analysis (IPA) results for candidate genes in the TSH and FT4 associated loci. A single protein network connects most of the identified loci.
(PDF)

Table S1 Analysis details and methods for individual GWAS studies.
(XLS)

Table S2 GWAS results of top SNPs for single cohorts. In the row named “G/I” we report the imputation quality (RSQR or INFO, according to study specific imputation method) or indicate with the letter “G” if the SNP was genotyped (G).
(XLSX)

Table S3 Heterogeneity analysis of South European vs North American cohorts. The table shows the results of the two meta-analyses carried out in studies of South Europe and North America individuals, respectively. For each meta-analysis, we report the frequency of the effect allele (FreqA1), the effect size and the corresponding standard error (Effect, StdErr), the combined association pvalue (P), the pvalue for heterogeneity between studies (Het P), and the number of samples analyzed (N). The last two columns report the pvalue for differences of the effect size estimated in the two groups, and the total number of samples analyzed.
(DOC)

Table S4 Association results for TSH and FT4 overlapping loci and their involvement in the negative feedback loop. The table shows the association results for all independent TSH (top panel) and FT4 (bottom panel) associated SNPs with FT4 and TSH levels, respectively. Effect sizes are standardized, so they represent the estimated phenotypic change, per each copy of the effect allele, in standard deviation units.
(DOC)

Table S5 Genotype risk score for TSH alleles in pregnant women.
(DOC)

Table S6 Association between TSH genetic risk score in pregnant women and subclinical hypothyroidism in pregnancy. Genotype risk score (GRS) was calculated in women with TSH level above reference range (>4.21 mIU/L) versus TSH level ≤4.21 mIU/L. Notably, excluding the 2 women with overt hypothyroidism (TSH>4.21 mIU/L and FT4 <9.13 pg/L), the results were essentially unchanged: Effect = 0.163, StdErr = 0.080, P = 0.043, OR = 1.18. StdErr, standard error, OR, odds ratio.
(DOC)

Table S7 Association of SNPs reported by Gudmundsson and colleagues in our dataset. The table shows the association results for SNPs reported by Gudmundsson and colleagues [32] with TSH and FT4 levels available in our data-set. When the same marker was not available, we reported a proxy (r^2^>0.8) and the relative r^2^. StdErr, standard error. Loci reaching genome-wide significance in our data set with either the same SNP or a proxy are highlighted in bold.
(DOC)

Text S1 Supplemental acknowledgments and funding information, cohort description, phenotyping, genotyping, analysis methods, supplemental references.
(DOC)
