# Supplementary figures and images for: A Meta-Analysis of Thyroid-Related Traits Reveals Novel Loci and Gender-Specific Differences in the Regulation of Thyroid Function
Source: PLoS Genet. 2013 Feb 7;9(2):e1003266. doi: 10.1371/journal.pgen.1003266 (PMC3567175; doi:10.1371/journal.pgen.1003266)

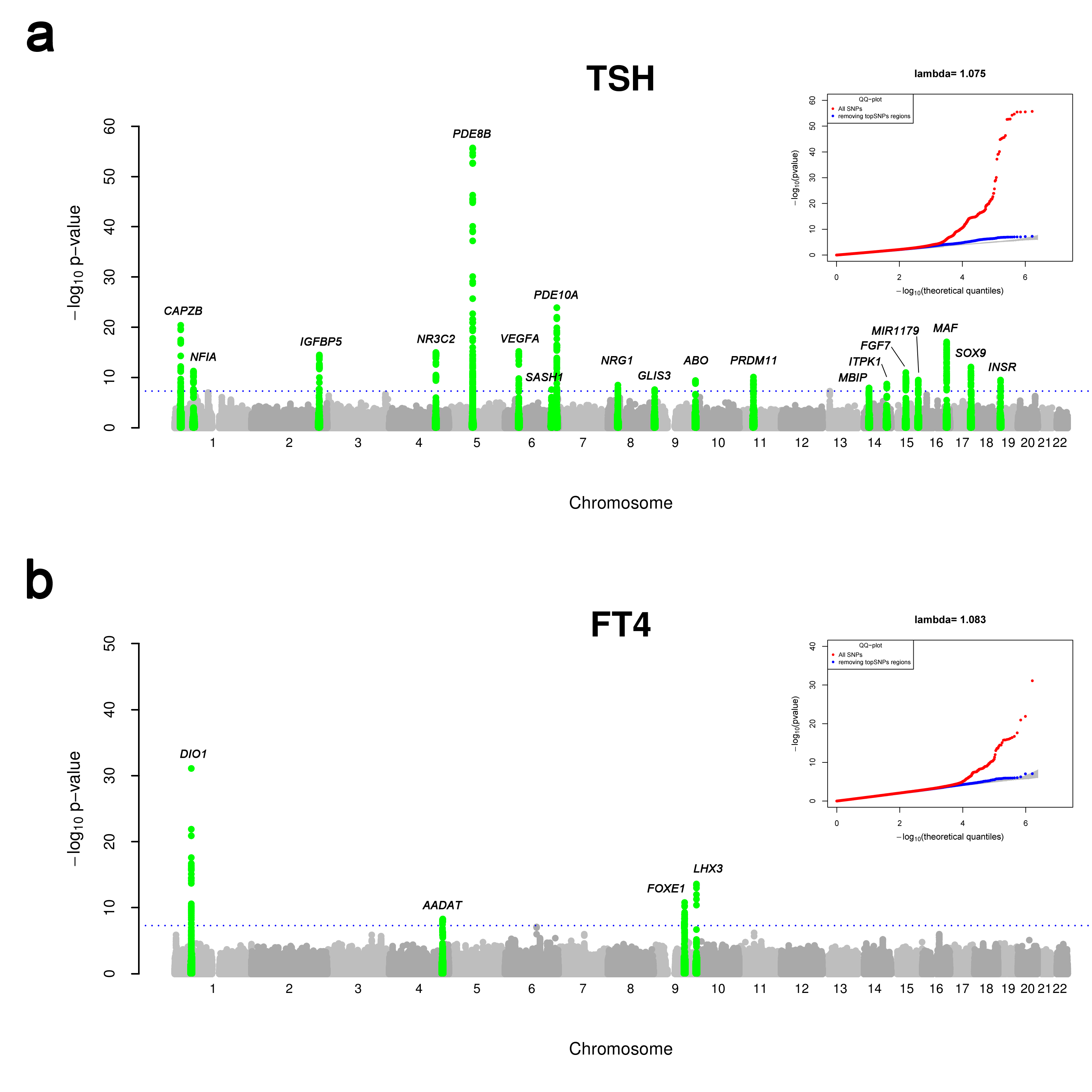

Supplement: Figure S1 — Manhattan plots from meta-analysis results of serum TSH (panel A) and FT4 (panel B) levels. SNPs are plotted on the x axis according to their position (build 36) on each chromosome against association with TSH (A) and FT4(B) on the y axis (shown as –log10 P value) in. The loci highlighted in green are those that reached genome-wide significance (P<5×10^−8^). In each panel, quantile-quantile plots obtained with all SNPs (red dots) and after removal of SNPs within associated regions (blue dots) are also shown. The gray area corresponds to the 90% confidence region from a null distribution of P values (generated from 100 simulations). (TIF) [file pgen.1003266.s001.tif]

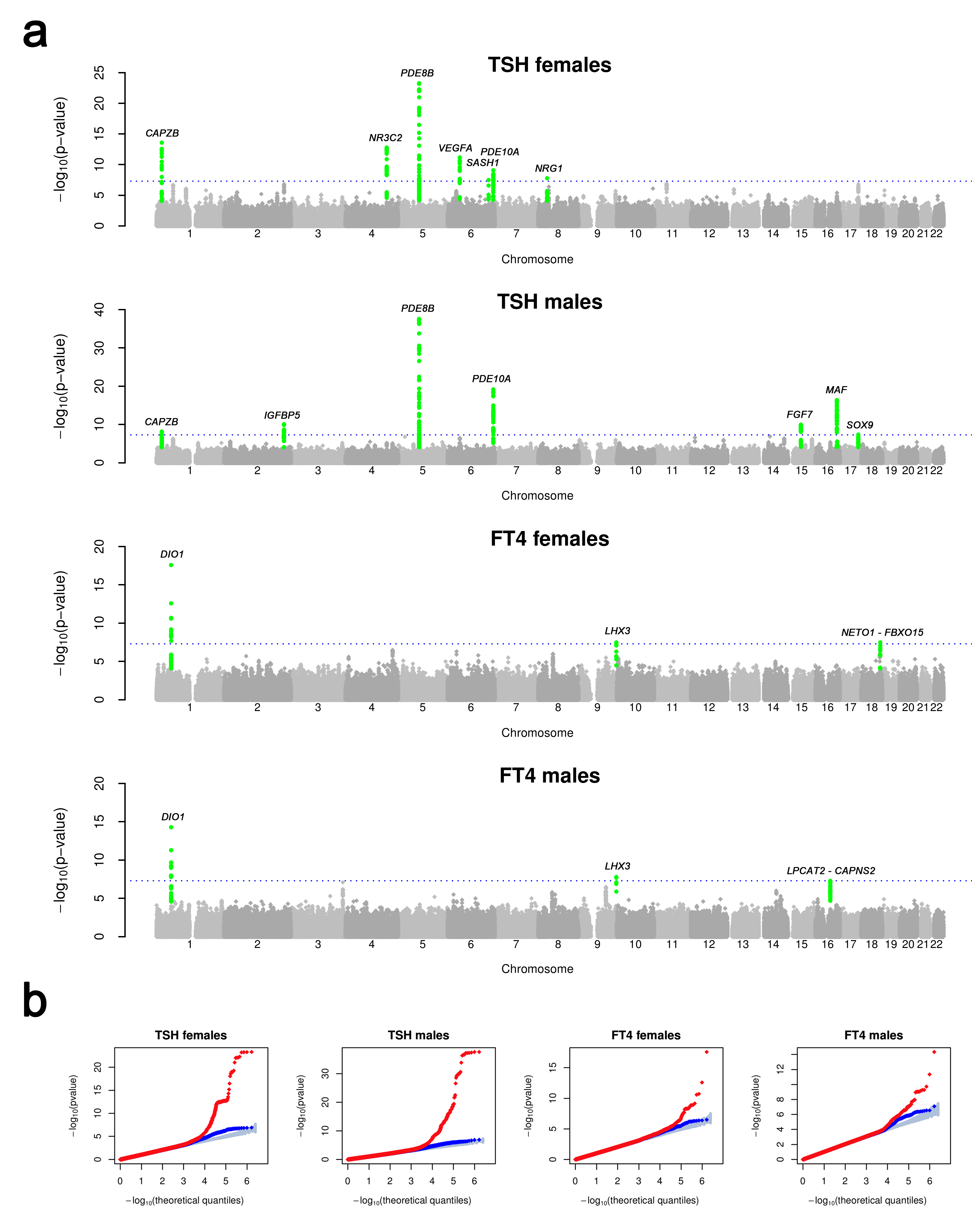

Supplement: Figure S2 — Panel A. Manhattan plots from meta-analysis results of serum TSH and FT4 levels for men and women separately are shown as indicated. SNPs are plotted on the x axis according to their position (build 36) on each chromosome; association with TSH and FT4 is indicated on the y axis (as –log10 P value). Signals reaching genome-wide statistical significance in the gender specific analysis are shown in green. Panel B. Quantile-quantile plots are shown for all SNPs (red dots) and after removal of SNPs within associated regions (blue dots). (TIF) [file pgen.1003266.s002.tif]
