# Supplementary material for: A Meta-Analysis of Thyroid-Related Traits Reveals Novel Loci and Gender-Specific Differences in the Regulation of Thyroid Function
Source: PLoS Genet. 2013 Feb 7;9(2):e1003266. doi: 10.1371/journal.pgen.1003266 (PMC3567175; doi:10.1371/journal.pgen.1003266)
Supplement: Figure S3 — Ingenuity pathway analysis (IPA) results for candidate genes in the TSH and FT4 associated loci. A single protein network connects most of the identified loci. (PDF) [file pgen.1003266.s003.pdf]

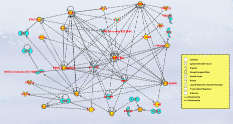

Blue color node genes are present in the TSH and FT4 list
Yellow color node genes are not present in the list

© 2000-2012 Ingenuity Systems, Inc. All rights reserved.
