# Supplementary material for: A Meta-Analysis of Thyroid-Related Traits Reveals Novel Loci and Gender-Specific Differences in the Regulation of Thyroid Function
Source: PLoS Genet. 2013 Feb 7;9(2):e1003266. doi: 10.1371/journal.pgen.1003266 (PMC3567175; doi:10.1371/journal.pgen.1003266)
Supplement: Table S3 — Heterogeneity analysis of South European vs North American cohorts. The table shows the results of the two meta-analyses carried out in studies of South Europe and North America individuals, respectively. For each meta-analysis, we report the frequency of the effect allele (FreqA1), the effect size and the corresponding standard error (Effect, StdErr), the combined association pvalue (P), the pvalue for heterogeneity between studies (Het P), and the number of samples analyzed (N). The last two columns report the pvalue for differences of the effect size estimated in the two groups, and the total number of samples analyzed. (DOC) [file pgen.1003266.s006.doc]

**Table S3. Heterogeneity analysis of South European vs North American cohorts.**

|  | | | | South Europeans (S.E.) | | | | | | North Americans (N.A.) | | | | | | S.E vs N.A. | |
| --- | --- | --- | --- | --- | --- | --- | --- | --- | --- | --- | --- | --- | --- | --- | --- | --- | --- |
| Gene | SNP | A1/A2 | FreqA1 | | Effect | StdErr | P | Het P | N | FreqA1 | Effect | StdErr | P | Het *P* | N | Het P | N |
| TSH levels |  |  |  | |  |  |  |  |  |  |  |  |  |  |  |  |  |
| *PDE8B* | rs6885099 | A/G | 0.5484 | | -0.1511 | 0.0182 | 1.12 X 10^-16^ | 0.125 | 7488 | 0.6125 | -0.1419 | 0.0200 | 1.17 X 10^-12^ | 0.799 | 5407 | 0.7353 | 12895 |
| *PDE10A* | rs753760 | C/G | 0.6949 | | 0.1058 | 0.0198 | 8.81 X 10^-08^ | 0.343 | 7488 | 0.6701 | 0.0737 | 0.0223 | 0.0009389 | 0.208 | 5407 | 0.2847 | 12895 |
| *CAPZB* | rs10799824 | A/G | 0.1707 | | -0.0869 | 0.0243 | 0.0003451 | 0.457 | 7488 | 0.1709 | -0.0818 | 0.0276 | 0.003078 | 0.004 | 5407 | 0.8904 | 12895 |
| *MAF/LOC440389* | rs3813582 | T/C | 0.6434 | | 0.1125 | 0.0191 | 4.17 X 10^-09^ | 0.296 | 7488 | 0.6452 | 0.0747 | 0.022 | 0.0006724 | 0.203 | 5407 | 0.1973 | 12895 |
| *VEGFA* | rs9472138 | T/C | 0.2714 | | -0.0807 | 0.0205 | 8.20 X 10^-05^ | 0.077 | 7488 | 0.2915 | -0.0903 | 0.0217 | 3.14 X 10^-05^ | 0.097 | 5407 | 0.7492 | 12895 |
| *VEGFA* | rs11755845 | T/C | 0.3185 | | -0.0746 | 0.0199 | 0.0001809 | 0.464 | 7488 | 0.2474 | -0.1134 | 0.0237 | 1.65 X 10^-06^ | 0.947 | 5407 | 0.2129 | 12895 |
| *NR3C2* | rs10032216 | T/C | 0.7660 | | 0.1069 | 0.022 | 1.20 X 10^-06^ | 0.577 | 7488 | 0.7923 | 0.0679 | 0.0246 | 0.00577 | 0.533 | 5407 | 0.2402 | 12895 |
| *IGFBP5* | rs13015993 | A/G | 0.7501 | | 0.0656 | 0.0207 | 0.001543 | 0.928 | 7488 | 0.7379 | 0.1042 | 0.0227 | 4.37 X 10^-06^ | 0.470 | 5407 | 0.2117 | 12895 |
| *NR3C2* | rs10032216 | T/C | 0.7660 | | 0.1069 | 0.022 | 1.20 X 10^-06^ | 0.577 | 7488 | 0.7923 | 0.0679 | 0.0246 | 0.00577 | 0.533 | 5407 | 0.2402 | 12895 |
| *SOX9* | rs9915657 | T/C | 0.6129 | | -0.0658 | 0.0188 | 0.0004785 | 0.224 | 7488 | 0.5473 | -0.0827 | 0.0202 | 4.22 X 10^-05^ | 0.964 | 5407 | 0.5427 | 12895 |
| *NFIA* | rs334699 | A/G | 0.0543 | | -0.1218 | 0.0423 | 0.003965 | 0.005 | 7488 | 0.0471 | -0.0588 | 0.048 | 0.2202 | 0.589 | 5407 | 0.3278 | 12895 |
| *FGF7* | rs10519227 | A/T | 0.2765 | | -0.0612 | 0.0207 | 0.003175 | 0.937 | 7488 | 0.2224 | -0.077 | 0.0249 | 0.001966 | 0.313 | 5407 | 0.6278 | 12895 |
| *PRDM11* | rs17723470 | T/C | 0.2052 | | -0.0551 | 0.025 | 0.0274 | 0.709 | 7488 | 0.2923 | -0.0691 | 0.0221 | 0.001782 | 0.646 | 5407 | 0.6764 | 12895 |
| *MIR1179* | rs17776563 | A/G | 0.3340 | | -0.1087 | 0.0198 | 3.76 X 10^-08^ | 0.901 | 7488 | 0.3488 | -0.049 | 0.0218 | 0.02484 | 0.225 | 5407 | 0.04392 | 12895 |
| *INSR* | rs4804416 | T/G | 0.5395 | | -0.0432 | 0.0187 | 0.02081 | 0.173 | 7488 | 0.5894 | -0.0605 | 0.0202 | 0.002797 | 0.142 | 5407 | 0.5322 | 12895 |
| ***ABO*** | **rs657152** | **A/C** | **0.3166** | | **0.1382** | **0.0196** | **1.919 X 10^-12^** | **0.001** | **7488** | **0.4100** | **0.0416** | **0.0205** | **0.04215** | **0.092** | **5407** | **0.00071** | **12895** |
| *ITPK1* | rs11624776 | A/C | 0.6104 | | -0.0937 | 0.0200 | 2.72 X 10^-06^ | 0.924 | 7488 | 0.6419 | -0.0447 | 0.0264 | 0.08989 | 0.378 | 5407 | 0.1418 | 12895 |
| *NRG1* | rs7825175 | A/G | 0.2009 | | -0.0735 | 0.0238 | 0.001987 | 0.8 | 7488 | 0.2338 | -0.033 | 0.0252 | 0.1918 | 0.754 | 5407 | 0.2454 | 12895 |
| *MBIP* | rs1537424 | T/C | 0.6301 | | -0.0444 | 0.0185 | 0.01646 | 0.195 | 7488 | 0.5899 | -0.0376 | 0.0213 | 0.0777 | 0.645 | 5407 | 0.8107 | 12895 |
| *SASH1* | rs9497965 | T/C | 0.4062 | | 0.0716 | 0.019 | 0.0001637 | 0.874 | 7488 | 0.4493 | 0.0077 | 0.0211 | 0.7155 | 0.514 | 5407 | 0.02531 | 12895 |
| *GLIS3* | rs1571583 | A/G | 0.2820 | | 0.0328 | 0.0200 | 0.1012 | 0.846 | 7488 | 0.2742 | 0.0911 | 0.0223 | 4.36 X 10^-05^ | 0.360 | 5407 | 0.05308 | 12895 |
| FT4 levels |  |  |  | |  |  |  |  |  |  |  |  |  |  |  |  |  |
| *DIO1* | rs2235544 | A/C | 0.4698 | | 0.0.1257 | 0.0.0214 | 4.24 X 10^-09^ | 0.0.198 | 6084 | 0.5046 | 0.0.1421 | 0.0.0313 | 5.52 X 10^-06^ | 0.0.354 | 2077 | 0.0.666 | 8161 |
| *LHX3* | rs7860634 | A/G | 0.4733 | | 0.0984 | 0.0215 | 4.5 X 10^-06^ | 0.229 | 6084 | 0.5557 | 0.0874 | 0.0315 | 0.005526 | 0.901 | 2077 | 0.774 | 8161 |
| *FOXE1* | rs7045138 | T/C | 0.5413 | | 0.0467 | 0.0337 | 0.1665 | 0.656 | 1997 | 0.5057 | 0.1415 | 0.0372 | 0.0001443 | 0.540 | 2077 | 0.059 | 4074 |
| *AADAT* | rs11726248 | A/G | 0.0913 | | 0.0.0971 | 0.0385 | 0.01159 | 0.927 | 6084 | 0.1100 | 0.0963 | 0.0494 | 0.05119 | 0.244 | 2077 | 0.990 | 8161 |
| *LPCAT2/CAPNS2* | rs6499766 | A/T | 0.4300 | | 0.088 | 0.0218 | 5.33 X 10^-05^ | 0.861 | 6084 | 0.5014 | -0.0198 | 0.0319 | 0.5354 | 0.447 | 2077 | 0.005 | 8161 |
| *NETO1/FBXO15* | rs7240777 | A/G | 0.5567 | | -0.041 | 0.0217 | 0.05939 | 0.512 | 6084 | 0.5617 | -0.0086 | 0.0329 | 0.7926 | 0.841 | 2077 | 0.412 | 8161 |
