# Supplementary material for: A Meta-Analysis of Thyroid-Related Traits Reveals Novel Loci and Gender-Specific Differences in the Regulation of Thyroid Function
Source: PLoS Genet. 2013 Feb 7;9(2):e1003266. doi: 10.1371/journal.pgen.1003266 (PMC3567175; doi:10.1371/journal.pgen.1003266)
Supplement: Table S4 — Association results for TSH and FT4 overlapping loci and their involvement in the negative feedback loop. The table shows the association results for all independent TSH (top panel) and FT4 (bottom panel) associated SNPs with FT4 and TSH levels, respectively. Effect sizes are standardized, so they represent the estimated phenotypic change, per each copy of the effect allele, in standard deviation units. (DOC) [file pgen.1003266.s007.doc]

**Table S4. Association results for TSH and FT4 overlapping loci and their involvement in the negative feedback loop.**

| TSH associated markers | | | **TSH** | | | **FT4** | | |
| --- | --- | --- | --- | --- | --- | --- | --- | --- |
| Gene | Marker Name | A1/A2 | Effect (StdErr) | P | N | Effect (StdErr) | P | N |
| ***PDE8B*** | **rs6885099** | **A/G** | **-0.141 (0.009)** | **1.95 X 10**^-56^ | **26042** | **0.025 (0.012)** | **0.034** | **17505** |
| *PDE10A* | rs753760 | C/G | 0.100 (0.010) | 1.21 X 10^-24^ | 25988 | -0.020 (0.013) | 0.114 | 17451 |
| *CAPZB* | rs10799824 | A/G | -0.113 (0.012) | 3.60 X 10^-21^ | 26031 | 0.020 (0.016) | 0.194 | 17494 |
| ***MAF/LOC440389*** | **rs3813582** | **T/C** | **0.082 (0.010)** | **8.45 X 10**^-18^ | **25948** | **-0.031 (0.013)** | **0.013** | **17411** |
| ***VEGFA*** | **rs9472138** | **T/C** | **-0.079 (0.010)** | **6.72 X 10**^-16^ | **25767** | **0.037 (0.013)** | **4.13 X 10**^-03^ | **17229** |
| *VEGFA* | rs11755845 | T/C | -0.065 (0.010) | 1.68 X 10^-10^ | 25710 | 0.019(0.013) | 0.153 | 17172 |
| *NR3C2* | rs10032216 | T/C | 0.087 (0.011) | 9.28 X 10^-16^ | 26053 | 0.014 (0.014) | 0.332 | 17516 |
| ***IGFBP5*** | **rs13015993** | **A/G** | **0.078 (0.010)** | **3.24 X 10**^-15^ | **26016** | **-0.026 (0.013)** | **0.047** | **17479** |
| *SOX9* | rs9915657 | T/C | -0.064 (0.009) | 7.53 X 10^-13^ | 25692 | 0.005 (0.012) | 0.642 | 17154 |
| ***NFIA*** | **rs334699** | **A/G** | **-0.141 (0.021)** | **5.40 X 10**^-12^ | **25757** | **0.053 (0.027)** | **0.048** | **17219** |
| *FGF7* | rs10519227 | A/T | -0.072 (0.011) | 1.02 X 10^-11^ | 25988 | 0.013 (0.014) | 0.352 | 17451 |
| *PRDM11* | rs17723470 | T/C | -0.065 (0.010) | 8,83 X 10^-11^ | 26054 | 0.005 (0.013) | 0.709 | 17517 |
| ***MIR1179*** | **rs17776563** | **A/G** | **-0.060 (0.010)** | **2.89 X 10**^-10^ | **25758** | **0.027 (0.013)** | **0.032** | **17221** |
| *INSR* | rs4804416 | T/G | -0.057 (0.009) | 3.16 X 10^-10^ | 25632 | 0.021 (0.012) | 0.076 | 17094 |
| *ABO* | rs657152 | A/C | 0.058 (0.009) | 4.11 X 10^-10^ | 25765 | -0.013 (0.012) | 0.271 | 17227 |
| *ITPK1* | rs11624776 | A/C | -0.064 (0.011) | 1.79 X 10^-9^ | 23482 | 0.011 (0.014) | 0.450 | 14945 |
| *NRG1* | rs7825175 | A/G | -0.066 (0.011) | 2.94 X 10^-9^ | 25996 | 0.020 (0.015) | 0.171 | 17459 |
| *ITPK1* | rs11624776 | A/C | -0.064 (0.011) | 1.79 X 10^-9^ | 23482 | 0.011 (0.014) | 0.450 | 14945 |
| *SASH1* | rs9497965 | T/C | 0.051 (0.009) | 2.25 X 10^-0^ | 25980 | -0.013 (0.012) | 0.277 | 17443 |
| ***GLIS3*** | **rs1571583** | **A/G** | **0.057 (0.010)** | **2.55 X 10**^-8^ | **25766** | **-0.028 (0.013)** | **0.040** | **17228** |
| FT4 associated markers | | | **FT4** | | | **TSH** | | |
| Gene | Marker Name | A1/A2 | Effect (StdErr) | P | N | Effect (StdErr) | P | N |
| *DIO1* | rs2235544 | A/C | 0.138 (0.012) | 7.87 X 10^-32^ | 17226 | -0.013 (0.009) | 0.136 | 25764 |
| ***LHX3*** | **rs7860634** | **A/G** | **0.102 (0.013)** | **2.30 X 10**^-14^ | **14529** | **0.031 (0.011)** | **5.25 X 10**^-3^ | **20933** |
| *AADAT* | rs11726248 | A/G | 0.111 (0.019) | 5.20 X 10^-9^ | 17515 | 0.014 (0.015) | 0.327 | 26052 |
| *FOXE1* | rs7045138 | T/C | 0.098 (0.015) | 1.50 X 10^-11^ | 10997 | 0.008 (0.010) | 0.451 | 19535 |
| *LPCAT2/CAPNS2* | rs6499766 | A/T | 0.056 (0.012) | 1.18 X 10^-6^ | 17489 | -0.009 **(**0.009**)** | 0.3374 | 26026 |
| *NETO1/FBXO15* | rs7240777 | A/G | -0.049 (0.012) | 3.13 X 10^-5^ | 17146 | 0.005 (0.009) | 0.559 | 25684 |
