# Supplementary material for: A Meta-Analysis of Thyroid-Related Traits Reveals Novel Loci and Gender-Specific Differences in the Regulation of Thyroid Function
Source: PLoS Genet. 2013 Feb 7;9(2):e1003266. doi: 10.1371/journal.pgen.1003266 (PMC3567175; doi:10.1371/journal.pgen.1003266)
Supplement: Table S5 — Genotype risk score for TSH alleles in pregnant women. (DOC) [file pgen.1003266.s008.doc]

**Table S5. Genotype risk score for TSH alleles in pregnant women.**

| **Model^a^** | **N** | **Beta^b^** | **SE** | **P-value** |
| --- | --- | --- | --- | --- |
| GRS^c^ | 858 | 0.109 | 0.015 | 3 X 10^-12^ |
| GRS in TPO-Ab negative women | 794 | 0.117 | 0.015 | 3 X 10^-14^ |
| GRS adjusted for fetal genotypes | 561 | 0.106 | 0.023 | 8 X 10^-6^ |

^a^ Linear regression of TSH level in pregnancy (inverse-normal transformation) against genotype risk score, with age and age-squared as covariates, excluding women on thyroid function medication and those of non-European descent.

^b^ beta per TSH-raising allele.

^c^ GRS= Genotype risk score was calculated as described in the Supplementary Methods in women with up to 2/9 SNPs missing.
