# Supplementary material for: A Meta-Analysis of Thyroid-Related Traits Reveals Novel Loci and Gender-Specific Differences in the Regulation of Thyroid Function
Source: PLoS Genet. 2013 Feb 7;9(2):e1003266. doi: 10.1371/journal.pgen.1003266 (PMC3567175; doi:10.1371/journal.pgen.1003266)
Supplement: Table S6 — Association between TSH genetic risk score in pregnant women and subclinical hypothyroidism in pregnancy. Genotype risk score (GRS) was calculated in women with TSH level above reference range (>4.21 mIU/L) versus TSH level ≤4.21 mIU/L. Notably, excluding the 2 women with overt hypothyroidism (TSH>4.21 mIU/L and FT4 <9.13 pg/L), the results were essentially unchanged: Effect = 0.163, StdErr = 0.080, P = 0.043, OR = 1.18. StdErr, standard error, OR, odds ratio. (DOC) [file pgen.1003266.s009.doc]

| **Model^a^** | **N** | **Effect^c^** | **StdErr** | **OR** | ***P*** |
| --- | --- | --- | --- | --- | --- |
| GRS^b^ | 858 | 0.162 | 0.078 | 1.176 | 0.039 |
| GRS in TPO-Ab negative women | 794 | 0.257 | 0.093 | 1.292 | 0.006 |

**Table S6. Association between TSH genetic risk score in pregnant women and subclinical hypothyroidism in pregnancy.**

^a^ Logistic regression of outcome against genotype risk score, with age and age-squared as covariates, excluding women taking thyroid function medication and those non-European descent.

^b^ GRS= Genotype risk score was calculated as described in the Supplementary Methods in women with up to 2/9 SNPs missing

^c^ beta per TSH-raising allele.
