# Supplementary material for: A Meta-Analysis of Thyroid-Related Traits Reveals Novel Loci and Gender-Specific Differences in the Regulation of Thyroid Function
Source: PLoS Genet. 2013 Feb 7;9(2):e1003266. doi: 10.1371/journal.pgen.1003266 (PMC3567175; doi:10.1371/journal.pgen.1003266)
Supplement: Table S7 — Association of SNPs reported by Gudmundsson and colleagues in our dataset. The table shows the association results for SNPs reported by Gudmundsson and colleagues [32] with TSH and FT4 levels available in our data-set. When the same marker was not available, we reported a proxy (r2>0.8) and the relative r2. StdErr, standard error. Loci reaching genome-wide significance in our data set with either the same SNP or a proxy are highlighted in bold. (DOC) [file pgen.1003266.s010.doc]

**Table S7.** **Association of SNPs reported by Gudmundsson and colleagues in our data-set.**

| **Marker Name** | **Marker Tested** | **chr** | **Nearest Gene** | **TSH** | | | | **FT4** | | | |
| --- | --- | --- | --- | --- | --- | --- | --- | --- | --- | --- | --- |
|  |  |  |  | **Effect** | **StdErr** | **P** | **N** | **Effect** | **StdErr** | **P** | **N** |
| **rs10799824** | **rs10799824** | **1** | ***CAPZB*** | **-0.113** | **0.012** | **3.60 X 10^-21^** | **26031** | **0.020** | **0.016** | **0.194** | **17494** |
| **rs334725** | **rs334725** | **1** | ***NFIA*** | **0.135** | **0.021** | **4.85 X 10**^-11^ | **25767** | **-0.053** | **0.027** | **0.050** | **17229** |
| rs17020124 | rs17020124 | 1 | *VAV3* | 0.081 | 0.017 | 9.95 X 10^-07^ | 26055 | -0.006 | 0.022 | 0.798 | 17518 |
| rs11694732 | rs11694732 | 2 | *TPO* | 0.027 | 0.009 | 0.00257 | 25968 | -0.027 | 0.012 | 0.022 | 17431 |
| **rs737308** | **rs737310 (r2=1)** | **2** | ***IGFBP5*** | **-0.078** | **0.010** | **1.14 X 10**^-14^ | **26013** | **0.027** | **0.013** | **0.038** | **17476** |
| rs966423 | rs966423 | 2 | *DIRC3* | 0.016 | 0.009 | 0.0726 | 25758 | -0.006 | 0.012 | 0.586 | 17220 |
| **rs10030849** | **rs11935941 (r2=0.99)** | **4** | ***NR3C2*** | **0.084** | **0.011** | **3.77 X 10^-15^** | **26052** | **0.010** | **0.014** | **0.492** | **17515** |
| **rs2046045** | **rs2046045** | **5** | ***PDE8B*** | **-0.142** | **0.009** | **2.14 X 10^-55^** | **25767** | **0.017** | **0.012** | **0.151** | **17229** |
| **rs729761** | **rs729761** | **6** | ***VEGFA*** | **-0.088** | **0.011** | **2.83 X 10**^-15^ | **23073** | **0.042** | **0.015** | **0.0038** | **14535** |
| **rs6923866** | **rs11755845 (r2=0.95)** | **6** | ***VEGFA*** | **-0.065** | **0.010** | **1.68 X 10^-10^** | **25710** | **0.019** | **0.013** | **0.153** | **17172** |
| **rs3008043** | **rs3008043** | **6** | ***PDE10A*** | **-0.092** | **0.010** | **5.23 X 10^-20^** | **21923** | **0.019** | **0.014** | **0.152** | **13386** |
| **rs2439302** | **rs2439302** | **8** | ***NRG1*** | **0.037** | **0.009** | **3.54 X 10^-05^** | **25932** | **-0.002** | **0.012** | **0.878** | **17395** |
| **rs965513** | **rs965513** | **9** | ***FOXE1*** | **-0.022** | **0.009** | **0.0196** | **25764** | **-0.068** | **0.012** | **3.45 X 10**^-08^ | **17226** |
| rs7913135 | rs7913750 (r2=1) | 10 | *NKX2-3* | -0.029 | 0.009 | 0.00122 | 26043 | -0.026 | 0.012 | 0.028 | 17506 |
| **rs7128207** | **rs7128207** | **11** | ***PRDM11*** | **0.050** | **0.009** | **3.05 X 10**^-08^ | **25756** | **-0.005** | **0.012** | **0.665** | **17218** |
| rs61938844 | NO PROXY | 12 | *ELK3* | ND | ND | ND | ND | ND | ND | ND | ND |
| rs944289 | rs944289 | 14 | *MBIP** | -0.043 | 0.009 | 2.22 X 10^-06^ | 25746 | 0.0211 | 0.012 | 0.077 | 17208 |
| rs116909374 | NO PROXY | 14 | *MBIP* | ND | ND | ND | ND | ND | ND | ND | ND |
| **rs34269820** | **rs957362 (r2=0.89)** | **14** | ***ITPK1*** | **-0.052** | **0.011** | **9.75 X 10^-07^** | **25766** | **0.0114** | **0.014** | **0.407** | **17228** |
| rs73362602 | not in HapMap2 | 14 | *SIVA1* | ND | ND | ND | ND | ND | ND | ND | ND |
| **rs73398284** | **rs17477923 (r2=0.95)** | **15** | ***FGF7*** | **0.068** | **0.010** | **1.40 X 10^-11^** | **26033** | **-0.022** | **0.013** | **0.099** | **17496** |
| **rs7190187** | **rs7188445 (r2=0.99)** | **16** | ***MAF/LOC440389*** | **-0.079** | **0.010** | **9.66 X 10**^-17^ | **25903** | **0.036** | **0.013** | **4.91 X 10**^-03^ | **17366** |
| **rs10420008** | **rs10420008** | **19** | ***INSR*** | **-0.074** | **0.012** | **7.23 X 10^-10^** | **21333** | **0.008** | **0.016** | **0.630** | **12795** |
| rs6082762 | rs2424440 (r2=1) | 20 | *FOXA2* | 0.031 | 0.011 | 4.08 X 10^-03^ | 26054 | 0.016 | 0.014 | 0.255 | 17517 |
