# Supplementary material for: A Meta-Analysis of Thyroid-Related Traits Reveals Novel Loci and Gender-Specific Differences in the Regulation of Thyroid Function
Source: PLoS Genet. 2013 Feb 7;9(2):e1003266. doi: 10.1371/journal.pgen.1003266 (PMC3567175; doi:10.1371/journal.pgen.1003266)
Supplement: Text S1 — Supplemental acknowledgments and funding information, cohort description, phenotyping, genotyping, analysis methods, supplemental references. (DOC) [file pgen.1003266.s011.doc]

**Text S1.**

**Supplemental acknowledgments and funding information.**

**BLSA**: We thank all study participants, volunteers, and study personnel that made this consortium possible. The BLSA was supported in part by the Intramural Research Program of the NIH, National Institute on Aging. A portion of that support was through a R&D contract with MedStar Research Institute.

**CHS**: This CHS research was supported by NHLBI contracts N01-HC-85239, N01-HC-85079 through N01-HC-85086; N01-HC-35129, N01 HC-15103, N01 HC-55222, N01-HC-75150, N01-HC-45133, HHSN268201200036C and NHLBI grants HL080295, HL087652, HL105756 with additional contribution from NINDS. Additional support was provided through AG-023629, AG-15928, AG-20098, and AG-027058 from the NIA. See also <http://www.chs-nhlbi.org/pi.htm>. DNA handling and genotyping was supported in part by National Center of Advancing Translational Technologies CTSI grant UL1TR000124 and National Institute of Diabetes and Digestive and Kidney Diseases grant DK063491 to the Southern California Diabetes Endocrinology Research Center and Cedars-Sinai Board of Governors' Chair in Medical Genetics (JIR).

**FHS**: The FHS research was conducted in part using data and resources from the Framingham Heart Study of the National Heart Lung and Blood Institute of the National Institutes of Health and Boston University School of Medicine. The analyses reflect intellectual input and resource development from the Framingham Heart Study investigators participating in the SNP Health Association Resource (SHARe) project. This work was partially supported by the National Heart, Lung and Blood Institute's Framingham Heart Study (Contract No. N01-HC-25195) and its contract with Affymetrix, Inc for genotyping services (Contract No. N02-HL-6-4278). A portion of this research utilized the Linux Cluster for Genetic Analysis (LinGA-II) funded by the Robert Dawson Evans Endowment of the Department of Medicine at Boston University School of Medicine and Boston Medical Center.

**GARP**: The GARP study would like to acknowledge support of the cooperating hospitals and referring rheumatologists, orthopaedic surgeons, and general practitioners in random order: Dr L N J E M Coene, department of orthopaedic surgery and Dr H K Ronday, department of rheumatology, Leyenburg Hospital, the Hague; I Speyer and Dr M L Westedt, department of rheumatology, Bronovo Hospital, the Hague; Dr D van Schaardenburg, department of rheumatology, Jan van Breemen Institute, Amsterdam; Dr A J Peeters and Dr D van Zeben, department of rheumatology, Reinier de Graaf Hospital, Delft; Dr E J Langelaan, department of orthopaedic surgery, Rijnland Hospital, Leiderdorp, and Dr Y Groeneveld, general practitioner, associated with the Leiden University Medical Centre. The Leiden University Medical Centre, the Dutch Arthritis Association and Pfizer Inc., Groton, CT, USA support the GARP study, whilst genotypic work was supported by the Netherlands Organization of Scientific Research (MW 904-61-095, 911-03-016, 917 66344 and 911-03-012), Leiden University Medical Centre, and by the “Centre of Medical System Biology” and the “Netherlands Consortium of Healthy Aging” in the framework of the Netherlands Genomics Initiative (NGI). Furthermore, the research leading to these results has received funding from the European Union's Seventh Framework Programme (FP7/2007-2011) under grant agreement n° 259679.

**HBCS**: The HBCS thanks all study participants as well as everybody involved in the Helsinki Birth Cohort Study. Helsinki Birth Cohort Study has been supported by grants from the Academy of Finland, the Finnish Diabetes Research Society, Folkhälsan Research Foundation, Novo Nordisk Foundation, Finska Läkaresällskapet, Signe and Ane Gyllenberg Foundation, University of Helsinki, European Science Foundation (EUROSTRESS), Ministry of Education, Ahokas Foundation, Emil Aaltonen Foundation, Juho Vainio Foundation, and Wellcome Trust (grant number WT089062).

**InCHIANTI**: The InCHIANTI study baseline (1998-2000) was supported as a "targeted project" (ICS110.1/RF97.71) by the Italian Ministry of Health and in part by the U.S. National Institute on Aging (Contracts: 263 MD 9164 and 263 MD 821336).

**LBC1921**: We thank the LBC1921 participants and team members. We thank Martha Pollard and Alison Pattie for data collection and data entry. The whole genome association study was funded by the Biotechnology and Biological Sciences Research Council (BBSRC). The LBC1921 data collection was funded by the BBSRC. The study was conducted within the University of Edinburgh Centre for Cognitive Ageing and Cognitive Epidemiology, supported by the BBSRC, Engineering and Physical Sciences Research Council (EPSRC), Economic and Social Research Council (ESRC), and Medical Research Council (MRC), as part of the cross-council Lifelong Health and Wellbeing Initiative. Lorna M Lopez is the beneficiary of a post-doctoral grant from the AXA Research Fund.

**LBC1936**: We thank the LBC1936 participants and team members. We thank Janie Corley, Caroline Brett, Caroline Cameron, Michelle Taylor, and Alison Pattie for data collection and data entry. The whole genome association study was funded by the Biotechnology and Biological Sciences Research Council (BBSRC). The LBC1936 research was supported by a programme grant from Research Into Ageing and continues with programme grants from Help the Aged/Research Into Ageing (Disconnected Mind). The study was conducted within the University of Edinburgh Centre for Cognitive Ageing and Cognitive Epidemiology, supported by the BBSRC, Engineering and Physical Sciences Research Council (EPSRC), Economic and Social Research Council (ESRC), and Medical Research Council (MRC), as part of the cross-council Lifelong Health and Wellbeing Initiative. Lorna M Lopez is the beneficiary of a post-doctoral grant from the AXA Research Fund.

**LifeLines**: The LifeLines Cohort Study, and generation and management of GWAS genotype data for the LifeLines Cohort Study is supported by the Netherlands Organization of Scientific Research NWO (grant 175.010.2007.006), the Economic Structure Enhancing Fund (FES) of the Dutch government, the Ministry of Economic Affairs, the Ministry of Education, Culture and Science, the Ministry for Health, Welfare and Sports, the Northern Netherlands Collaboration of Provinces (SNN), the Province of Groningen, University Medical Center Groningen, the University of Groningen, Dutch Kidney Foundation and Dutch Diabetes Research Foundation. We thank Behrooz Alizadeh, Annemieke Boesjes, Marcel Bruinenberg, Noortje Festen, Ilja Nolte, Lude Franke, Mitra Valimohammadi for their help in creating the GWAS database, and Rob Bieringa, Joost Keers, René Oostergo, Rosalie Visser, Judith Vonk for their work related to data-collection and validation. The authors are grateful to the study participants, the staff from the LifeLines Cohort Study and Medical Biobank Northern Netherlands, and the participating general practitioners and pharmacists.

**LLS**: We thank all participants of the Leiden Longevity Study. The research leading to these results has received funding from the European Union's Seventh Framework Programme (FP7/2007-2011) under grant agreement no. 259679. The Leiden Longevity Study is supported by a grant from the Innovation-Oriented Research Program on Genomics (SenterNovem IGE05007), the Centre for Medical Systems Biology, and the Netherlands Consortium for Healthy Ageing (grant 050-060-810), all in the framework of the Netherlands Genomics Initiative, Netherlands Organization for Scientific Research (NWO).

**MICROS**: For the MICROS study, we thank the primary care practitioners in the villages of the Val Venosta and the personnel of the Hospital of Silandro (Department of Laboratory Medicine) for their participation and collaboration in the research project. In South Tyrol, the study was supported by the Ministry of Health and Department of Educational Assistance, University and Research of the Autonomous Province of Bolzano, and the South Tyrolean Sparkasse Foundation.

**NBS**: The NBS thanks the individuals that participated in the study and whose contribution made this work possible. Collection of samples and data in the Netherlands was funded in part by a research investment grant of the Radboud University Nijmegen Medical Centre and by the National Computing Facilities Foundation (NCF) for the use of supercomputer facilities, with financial support from the NWO.

**OOA**: The Old Order Amish study wish to thank the Amish community for their cooperation and partnership in research and the personnel at the Amish Research Clinic for their extraordinary efforts. The work with the Amish was supported by research grants U01 HL72515 and R01 AG18728 and by the University of Maryland General Clinical Research Center, grant M01 RR 16500; the Johns Hopkins University General Clinical Research Center, grant M01 RR 000052; General Clinical Research Centers Program, National Center for Research Resources (NCRR), NIH; and the Baltimore Veterans Administration Geriatric Research and Education Clinical Center (GRECC).

**PROSPER/PHASE**: The PROSPER study was supported by an investigator initiated grant obtained from Bristol-Myers Squibb. Prof. Dr. J. W. Jukema is an Established Clinical Investigator of the Netherlands Heart Foundation (grant 2001 D 032). Support for genotyping was provided by the seventh framework program of the European commission (grant 223004) and by the Netherlands Genomics Initiative (Netherlands Consortium for Healthy Aging grant 050-060-810).

**RS**: The generation and management of GWAS genotype data for the Rotterdam Study is supported by the Netherlands Organisation of Scientific Research NWO Investments (nr. 175.010.2005.011, 911-03-012). This study is funded by the Research Institute for Diseases in the Elderly (014-93-015; RIDE2), the Netherlands Genomics Initiative (NGI)/Netherlands Organisation for Scientific Research (NWO) project nr. 050-060-810. We thank Pascal Arp, Mila Jhamai, Marijn Verkerk, Lizbeth Herrera and Marjolein Peters for their help in creating the GWAS database, and Karol Estrada and Maksim V. Struchalin for their support in creation and analysis of imputed data. The Rotterdam Study is funded by Erasmus Medical Center and Erasmus University, Rotterdam, Netherlands Organization for the Health Research and Development (ZonMw), the Research Institute for Diseases in the Elderly (RIDE), the Ministry of Education, Culture and Science, the Ministry for Health, Welfare and Sports, the European Commission (DG XII), and the Municipality of Rotterdam. The authors are grateful to the study participants, the staff from the Rotterdam Study and the participating general practitioners and pharmacists. We would like to thank Karol Estrada, Dr. Fernando Rivadeneira, Dr. Tobias A. Knoch, Anis Abuseiris, Luc V. de Zeeuw, and Rob de Graaf (Erasmus MC Rotterdam, The Netherlands) for their help in creating GRIMP, and BigGRID, MediGRID, and Services@MediGRID/D-Grid (funded by the German Bundesministerium fuer Forschung und Technology; grants 01 AK 803 A-H, 01 IG 07015 G) for access to their grid computing resources.

**SardiNIA**: The SardiNIA study thanks the many individuals who generously participated in this study, Monsignore Piseddu, Bishop of Ogliastra, the mayors and citizens of the Sardinian towns (Lanusei, Ilbono, Arzana, and Elini), and the head of the Public Health Unit ASL4 for their volunteerism and cooperation; the team also thanks the physicians Marco Orrù, Maria Grazia Pilia, Liana Ferreli, Francesco Loi, nurses Paola Loi, Monica Lai and Anna Cau who carried out participant physical exams, the recruitment personnel Susanna Murino, Michele Marongiu for informatic support. We also thank Fabio Busonero, Antonella Mulas, Mariano Dei, Sandra Lai, Andrea Maschio for genotyping and Monia Lobina for DNA and serum extraction. This work was supported by the Intramural Research Program of the National Institute on Aging (NIA), National Institutes of Health (NIH), with contracts NO1-AG-1–2109 and HHSN271201100005C; by Italian grants FISM 2011/R/13 “Approccio razionale per la ricerca di composti per la cura della sclerosi multipla basato sull’analisi dei target biologici individuati dagli studi di associazione sull’intero genoma in Sardegna”, FaReBio2011 “Farmaci e Reti Biotecnologiche di Qualità”, Funds MIUR/CNR for rare diseases and molecular screening, PNR-CNR Aging Program 2012-2014. The efforts of SS were supported in part by contract 263-MA-410953 from the NIA to the University of Michigan and by research grant HG002651 and HL084729 from the NIH.

**TwinsUK**: The TwinsUK study is grateful to the volunteer twins who made available their time. We thank the staff from the genotyping facilities at the Wellcome Trust Sanger Institute and the Center for Inherited Disease Research as part of a National Eye Institute/National Institutes of Health project grant. We gratefully acknowledge the contribution of Abbott Diagnostics, North Ryde, Australia, which provided support for the biochemical analysis. This study received funding from the Wellcome Trust; the European Community’s Seventh Framework Program grant agreement (FP7/2007-2013); ENGAGE project grant agreement (HEALTH-F4-2007-201413); the Department of Health via the National Institute for Health Research (NIHR) Comprehensive Biomedical Research Centre award to Guy’s & St Thomas’ NHS Foundation Trust in partnership with King’s College London; the Canadian Institutes of Health Research, Canadian Foundation for Innovation, Fonds de la Recherche en Santé Québec, Ministère du Développement Économique, de l’Innovation et de l’Exportation Québec and the Lady Davis Institute of the Jewish General Hospital (JBR); the Australian National Health and Medical Research Council (Project Grants 1010494, 1031422) and the Sir Charles Gairdner Hospital Research Fund (JW, SGW). N.S.’s research is supported by the Wellcome Trust (grant codes WT098051 and WT091310), the EU FP7 (EPIGENESYS grant code 257082 and BLUEPRINT grant code HEALTH-F5-2011-282510). T.D.S. is an NIHR senior investigator.

**ValBorbera**: The ValBorbera study thanks the inhabitants of the Val Borbera for participating to the study, and the local administrations and the ASL-Novi Ligure for support. The research was supported by funds from Compagnia di San Paolo, Torino, Italy; Fondazione Cariplo, Italy and Ministry of Health, Ricerca Finalizzata 2008.

**EFSOCH**: The Exeter Family Study of Childhood Health (EFSOCH) was supported by South West NHS Research and Development, Exeter NHS Research and Development, the Darlington Trust, and the Peninsula NIHR Clinical Research Facility at the University of Exeter. Genotyping of EFSOCH DNA samples was supported by the Endocrine Research Fund. A.T.H. and B.M.S. are employed as core members of the Peninsula NIHR Clinical Research Facility. R.M.F. is funded by a Sir Henry Wellcome Postdoctoral Fellowship (Wellcome Trust grant: 085541/Z/08/Z). We are extremely grateful to the EFSOCH study participants and the study team. The opinions given in this paper do not necessarily represent those of NIHR, the NHS or the Department of Health.

**Cohort description**

The following cohorts are part of the Meta-Thyroid consortium:

**Baltimore longitudinal study on Aging (BLSA)**: BLSA is a population-based study aimed to evaluate contributors of healthy aging in the older population residing predominantly in the Baltimore-Washington DC area [1]. Starting in 1958, participants are examined every one to four years depending on their age. Currently there are approximately 1100 active participants enrolled in the study. The BLSA has continuing approval from the Institutional Review Board (IRB) of Medstar Research Institute.

**Cardiovascular Health Study (CHS):** CHS is a population-based cohort study of risk factors for coronary heart disease and stroke in adults aged 65 years or older conducted across four field centers in the United States [2]. The original cohort of 5,201 persons consisting of 4,925 Caucasians was recruited in 1989-1990 from a random sample of people on Medicare eligibility lists. An additional 687 African-Americans were enrolled subsequently. African American participants were excluded from this analysis of individuals of European ancestry. CHS participants completed standardized clinical examinations and questionnaires at study baseline and at nine annual follow-up visits. DNA was extracted from blood samples drawn on all participants who consented to genetic testing at the 1989-90 examination.

**Framingham Heart Study (FHS):** The original cohort of FHS was recruited in 1953 in the town of Framingham in Massachusetts. In 1971, an Offspring cohort of 5,124 (2,483 men with average age of 37 and 2,641 women with average age of 36) was recruited and has been examined every four years. The subjects in the Offspring cohort include adult children of Original cohort and spouses of offspring. All participants provided a written informed consent and all study protocols were approved by Boston University [3,4].

**Genetics, Arthrosis, and Progression study (GARP)**: The GARP study has been described in detail previously [5]. It aimed at identifying determinants of osteoarthritis and the progression of this disease. The study is based on sibships of white Dutch ancestry with clinical- and radiographically-confirmed osteoarthritis at two or more joint sites of the hand, spine (cervical or lumbar), knee or hip. In the current analyses we included 359 subjects from whom we had genome wide scan data and thyroid levels available.

**Helsinki Birth Cohort Study (HBCS)**: The HBCS is composed of 8,760 individuals born between the years 1934-44 in one of the two main maternity hospitals in Helsinki, Finland. Between 2001 and 2003, a randomly selected sample of 928 males and 1,075 females participated in a clinical follow-up study with a focus on cardiovascular, metabolic and reproductive health, cognitive function and depressive symptoms. Detailed information on the selection of the HBCS participants and on the study design can be found elsewhere [6]. Research plan of the HBCS was approved by the Institutional Review Board of the National Public Health Insitute and all participants have signed an informed consent.

**Invecchiare in Chianti** study (InCHIANTI): The InCHIANTI study is a population-based epidemiological study aimed at evaluating the factors that influence mobility in the older population living in the Chianti region in Tuscany, Italy. The details of the study have been previously reported [7,8]. Briefly, 1616 residents were selected from the population registry of Greve in Chianti (a rural area: 11,709 residents with 19.3% of the population > 65 years of age), and Bagno a Ripoli (Antella village near Florence; 4,704 inhabitants, with 20.3% of the population > 65 years of age). The participation rate was 90% (n=1453), and the subjects ranged between 21-102 years of age.

**LBC1921**: The LBC1921 cohort consists of 550 relatively healthy individuals, 316 females and 234 males, assessed on cognitive and medical traits at around 79 years of age. They were born in 1921, most took part in the Scottish Mental Survey of 1932, and almost all lived independently in the Lothian region (Edinburgh City and surrounding area) in Scotland. When tested, the sample had a mean age of 79.1 years (SD = 0.6). A full description of participant recruitment and testing can be found elsewhere [9]. Ethics permission for the study was obtained from the Multi-Centre Research Ethics Committee for Scotland (MREC/01/0/56) and from Lothian Research Ethics Committee (LREC/1998/4/183). The research was carried out in compliance with the Helsinki Declaration. All subjects gave written, informed consent.

**LBC1936:** The LBC1936 consists of 1,091 relatively healthy individuals assessed on cognitive and medical traits at around 70 years of age. They were born in 1936, most took part in the Scottish Mental Survey of 1947, and almost all lived independently in the Lothian region of Scotland. The sample of 548 men and 543 women had a mean age 69.6 years (SD = 0.8). A full description of participant recruitment and testing can be found elsewhere [10]. Ethics permission for the study was obtained from the Multi-Centre Research Ethics Committee for Scotland (MREC/01/0/56) and from Lothian Research Ethics Committee (LREC/2003/2/29). The research was carried out in compliance with the Helsinki Declaration. All subjects gave written, informed consent.

**LifeLines:** The LifeLines Cohort Study is a multi-disciplinary prospective population-based cohort study examining in a unique three-generation design the health and health-related behaviours of 165,000 persons living in the North East region of The Netherlands [11]. It employs a broad range of investigative procedures in assessing the biomedical, socio-demographic, behavioural, physical and psychological factors which contribute to the health and disease of the general population, with a special focus on multimorbidity. All survey participants are between 18 and 90 years old at the time of enrollment. Recruitment has been going on since the end of 2006, and until January 2011 over 40,000 participants have been included.

**Leiden Longevity Study (LLS):** For the Leiden Longevity Study, long-lived siblings of European descent were recruited together with their offspring and the partners of the offspring. Families were recruited if at least two long-lived siblings were alive and fulfilled the age criterion of 89 years or older for males and 91 years or older for females, representing less than 0.5% of the Dutch population in 2001 [12]. In total 944 long-lived siblings were included with a mean age of 94 years (range 89-104), 1671 offspring (61 years, 39-81) and 744 partners (60 years, 36-79). DNA from the Leiden Longevity Study was extracted from samples at baseline using conventional methods [13].

**MICROS:** The *MICROS* study is part of the genomic health care program 'GenNova' and was carried out in three villages of the Val Venosta, South Tyrol (Italy), in 2001-2003. It comprised members of the populations of Stelvio, Vallelunga and Martello. A detailed description of the *MICROS* study is available elsewhere [14]. Briefly, study participants were volunteers from three isolated villages located in the Italian Alps, in a German-speaking region bordering with Austria and Switzerland. Owing to geographical, historical and political reasons, the entire region experienced a prolonged period of isolation from surrounding populations. The study participants are connected among each other in a unique genealogy for the three villages. Information on the participant’s health status was collected through a standardized questionnaire.

**Nijmegen Biomedical Study (NBS):** Details of the NBS have been described before [15]. In brief, the Nijmegen Biomedical Study is a population- based cross-sectional study conducted by the Radboud University Nijmegen Medical Centre. Approval to conduct the study was obtained from the Institutional Review Board. Nijmegen is a town in the eastern part of The Netherlands with 156,000 inhabitants, approximately 87% of Caucasian descent. Age and sex stratified randomly selected adult (age 18 years and older) inhabitants of Nijmegen (N=22,452) received an invitation to fill out a postal questionnaire on lifestyle and medical history. A total of 6,434 participants donated blood for measurement of thyroid function and DNA-isolation. Informed consent was obtained from each participant, and the Institutional Review Board of the Radboud University Nijmegen Medical Centre approved the study.

**Old Order Amish (OOA)**

The Old Order Amish (OOA) study participants reported here were from ongoing studies of cardiovascular disease and longevity [16,17]. Nearly all of the enrolled individuals are descendants of a small number of Amish who settled in Lancaster County, Pennsylvania, in the mid-eighteenth century. A total of 1,136 individuals from these two studies had serum TSH measured by Immulite 2000 (Siemens), using a standardized third generation assay, and were previously genotyped with the 500K Affymetrix Mapping Array set. This study was approved by the Institutional Review Boards of the University of Maryland and the National Cancer Institute.

**PROSPER/PHASE**: All data come from the PROspective Study of Pravastatin in the Elderly at Risk (PROSPER). A detailed description of the study has been published elsewhere [18,19]. PROSPER was a prospective multicenter randomized placebo-controlled trial to assess whether treatment with pravastatin diminishes the risk of major vascular events in elderly. Between December 1997 and May 1999, we screened and enrolled subjects in Scotland (Glasgow), Ireland (Cork), and the Netherlands (Leiden). Men and women aged 70-82 years were recruited if they had pre-existing vascular disease or increased risk of such disease because of smoking, hypertension, or diabetes. A total number of 5,804 subjects were randomly assigned to pravastatin or placebo. A large number of prospective tests were performed including Biobank tests and cognitive function measurements.

**Rotterdam Study (RS):** The RS is a prospective population-based cohort study on determinants of chronic diseases in the elderly, which has been described previously [20,21]. The study comprised 7983 men and women aged 55 years and over, living in a district of Rotterdam, The Netherlands. Informed consent was obtained from each participant, and the Medical Ethics Committee of the Erasmus Medical Center Rotterdam approved the study.

**SardiNIA:** The SardiNIA study consists of 6,148 volunteers, males and females, ages 14–102 yr, recruited and phenotyped from a cluster of four towns in the Ogliastra province of Sardinia [22,23]. The local ethical committee approved the study protocol and all participants provided a written informed consent.

**Twins UK:** The Twins UK cohort consists of 2,217 female twins of northern European/UK ancestry (1,831 dizygotic and 386 monozygotic), aged 18–82 yr, from St Thomas’ UK Adult Twin Registry (TwinsUK), a volunteer sample recruited in the United Kingdom without selection for particular traits ([www.twinsuk.ac.uk/](http://www.twinsuk.ac.uk/)) [24].

**ValBorbera (INGI):** The Val Borbera (INGI) population is a collection of 1,664 genotyped samples collected in the Val Borbera Valley, a geographically isolated valley located within the Appennine Mountains in NorthWest Italy [25]. The valley is inhabited by about 3000 descendants from the original population, living in 7 villages along the valley and in the mountains. The valley was inhabited by about 10,000 people in the 19th century when endogamy was >80%. Participants were healthy people between 18 and 102 years of age that had at least one grandfather living in the valley.

**Genotyping and imputation**

Nine different genotyping platforms were used by the cohorts included in this meta-analysis: Illumina HumanHap 610K (HBCS, LBC1921, LBC1936), Illumina HumanHap 550K (BLSA, InCHIANTI, RS), Illumina HumanHap 660K (LLS, PROSPER), Illumina HumanHap 370K (CHS, NBS, ValBorbera), Illumina Cyto-SNP12 v2- 300K (LifeLines), Illumina HumanHap 300K (MICROS), the Affymetrix 500K in combination with the 10K supplemental array (SardiNIA), the Affymetrix 500K in combination with the 50K supplemental array (FHS), the Illumina HumanHap 300K, 550K and 610K arrays (TwinsUK). Each study performed genotyping quality control checks based on duplicate sample genotyping, SNP call rate, Hardy-Weinberg equilibrium, Mendelian inconsistencies, sex mismatch, and principle components methods were used to evaluate the presence of population stratification. Each study imputed 2.5 million HapMap SNPs for each participant using currently available imputation methods. BLSA, FHS, HBCS, InCHIANTI, LBC1921, LBC1936, MICROS, PROSPER, RS, SardiNIA, and ValBorbera used the MACH algorithm (http://www.sph.umich.edu/csg/abecasis/MaCH/); LifeLines, LLS and TwinsUK used IMPUTE (http://www.stats.ox.ac.uk/~marchini/software/gwas /impute), and CHS used BimBam (Servin, B. and Stephens, M. [Imputation-based analysis of association studies: candidate genes and quantitative traits.](http://stephenslab.uchicago.edu/MSpapers/Servin2007.pdf) PLoS Genetics, 2007). Further details are summarized in Table S1.

**Thyroid function measurements**

Methods used by each study cohort to measure TSH and FT4 levels are reported in Table S1.

**Statistical analyses**

All cohorts excluded subjects with thyroid medication, thyroid surgery, or with out-of-range TSH values (TSH > 4.0 mIU/L or TSH < 0.4 mIU/L). Linear regression analyses (additive model) were performed after applying inverse normal transformation to both TSH and FT4. Age, age-squared, and gender were used as covariates, as well as principal components axes or additional variables, when needed. Further details are summarized in Table S1.

**Association of TSH SNPs in pregnant women.**

Association of TSH lead SNPs was tested in pregnant women of the Exeter Family Study of Childhood Health (EFSOCH). EFSOCH is a consecutive birth cohort consisting of children born between 2000 and 2004 in central Exeter, UK, and their parents [26]. Both parents attended a study visit at 28 weeks of gestation, at which DNA was collected and a fasting blood sample was taken for biochemical assays. The local research ethics committees approved the study, and all adult participants gave informed written consent. Serum TSH and FT4 levels were determined in 974 pregnant women using an electrochemiluminescent immunoassay, run on the Modular E170 Analyzer (Roche, Burgess Hill, UK). The manufacturer’s population reference ranges (for non-pregnant samples) were: TSH, 0.35–4.5 mIU/L; and FT4, 11–24 pmol/L. For the analyses of the pregnant women, we used reference ranges specific for the assay and 28th week of gestation based on our own set of TPOAb-negative, healthy, pregnant women (n=901): TSH, 0.49–4.21 mIU/L and FT4, 9.13–15.17 pmol/L [27]. Serum TPOAb levels were determined using the competitive immunoassay (Roche) in 970 pregnant women. A titer above 34 IU/mL was considered positive.

DNA samples were genotyped at KBiosciences (Hoddesdon, UK; www.kbioscience.co.uk), using their own system of fluorescence-based competitive allele-specific PCR (KASPar). Call rates of the 9 SNPs analyzed were >95% and there was no evidence of deviation from Hardy-Weinberg equilibrium (P > 0.05). Concordance between duplicate samples (10% of total) was >99% for all SNPs.

Association analyses were carried out using Stata SE v.10 (StataCorp, Texas, USA). A total of 862 pregnant women with TSH levels and genotype were available for analysis (mean age 30.4 years, s.d. 5.3 years). Women taking medication for thyroid disorders were excluded (*n* = 14). We used linear regression to analyse the association between TSH level (inverse normal transformation) and each individual SNP (coded as 0, 1 or 2 TSH-increasing alleles), with age and age-squared as covariables. We then constructed a genetic risk score (GRS) for all women with at least 7 available SNPs:

GRS = weighted score x N available SNPs / sum of weights of available SNPs,

where

weighted score = *w1* x SNP*1* + *w2* x SNP*2* +…*wi* x SNP*i*

and *wi* is the beta coefficient from the association between TSH levels and SNP*i.* We performed linear regression of TSH level against the GRS (additive model), with age and age-squared as covariables. We performed all analyses twice: first including, and then excluding the women who tested positive for TPO antibodies (7.5% of the sample). We also verified that the results were not materially altered on adjustment for fetal genotype at all 9 SNPs. Finally, we used logistic regression to assess the association between subclinical hypothyroidism in pregnancy and the GRS, again adjusting for age and age-squared. We defined cases as TSH > 4.21 mIU/L (the upper limit of the reference range) and controls as TSH <=4.21 mIU/L [27].

**Supplemental references**

1. Shock NW, Greulich, R.C., Costa, P.T., Andres, R., Lakatta, E.G., Arenberg. D., Tobin, J.D. (1984) Normal Human Aging: The Baltimore Study of Aging. US Government Printing Office; Washington, DC.

2. Fried LP, Borhani NO, Enright P, Furberg CD, Gardin JM, et al. (1991) The Cardiovascular Health Study: design and rationale. Ann Epidemiol 1: 263-276.

3. Splansky GL, Corey D, Yang Q, Atwood LD, Cupples LA, et al. (2007) The Third Generation Cohort of the National Heart, Lung, and Blood Institute's Framingham Heart Study: design, recruitment, and initial examination. Am J Epidemiol 165: 1328-1335.

4. Fox CS, Pencina MJ, D'Agostino RB, Murabito JM, Seely EW, et al. (2008) Relations of thyroid function to body weight: cross-sectional and longitudinal observations in a community-based sample. Arch Intern Med 168: 587-592.

5. Riyazi N, Meulenbelt I, Kroon HM, Ronday KH, Hellio le Graverand MP, et al. (2005) Evidence for familial aggregation of hand, hip, and spine but not knee osteoarthritis in siblings with multiple joint involvement: the GARP study. Ann Rheum Dis 64: 438-443.

6. Eriksson JG (2011) Early growth and coronary heart disease and type 2 diabetes: findings from the Helsinki Birth Cohort Study (HBCS). Am J Clin Nutr 94: 1799S-1802S.

7. Ferrucci L, Bandinelli S, Benvenuti E, Di Iorio A, Macchi C, et al. (2000) Subsystems contributing to the decline in ability to walk: bridging the gap between epidemiology and geriatric practice in the InCHIANTI study. J Am Geriatr Soc 48: 1618-1625.

8. Melzer D, Perry JR, Hernandez D, Corsi AM, Stevens K, et al. (2008) A genome-wide association study identifies protein quantitative trait loci (pQTLs). PLoS Genet 4: e1000072.

9. Deary IJ, Whiteman MC, Starr JM, Whalley LJ, Fox HC (2004) The impact of childhood intelligence on later life: following up the Scottish mental surveys of 1932 and 1947. J Pers Soc Psychol 86: 130-147.

10. Deary IJ, Gow AJ, Taylor MD, Corley J, Brett C, et al. (2007) The Lothian Birth Cohort 1936: a study to examine influences on cognitive ageing from age 11 to age 70 and beyond. BMC Geriatr 7: 28.

11. Stolk RP, Rosmalen JG, Postma DS, de Boer RA, Navis G, et al. (2008) Universal risk factors for multifactorial diseases: LifeLines: a three-generation population-based study. Eur J Epidemiol 23: 67-74.

12. Schoenmaker M, de Craen AJ, de Meijer PH, Beekman M, Blauw GJ, et al. (2006) Evidence of genetic enrichment for exceptional survival using a family approach: the Leiden Longevity Study. Eur J Hum Genet 14: 79-84.

13. Beekman M, Blauw GJ, Houwing-Duistermaat JJ, Brandt BW, Westendorp RG, et al. (2006) Chromosome 4q25, microsomal transfer protein gene, and human longevity: novel data and a meta-analysis of association studies. J Gerontol A Biol Sci Med Sci 61: 355-362.

14. Pattaro C, Marroni F, Riegler A, Mascalzoni D, Pichler I, et al. (2007) The genetic study of three population microisolates in South Tyrol (MICROS): study design and epidemiological perspectives. BMC Med Genet 8: 29.

15. Hoogendoorn EH, Hermus AR, de Vegt F, Ross HA, Verbeek AL, et al. (2006) Thyroid function and prevalence of anti-thyroperoxidase antibodies in a population with borderline sufficient iodine intake: influences of age and sex. Clin Chem 52: 104-111.

16. Sorkin J, Post W, Pollin TI, O'Connell JR, Mitchell BD, et al. (2005) Exploring the genetics of longevity in the Old Order Amish. Mech Ageing Dev 126: 347-350.

17. Mitchell BD, McArdle PF, Shen H, Rampersaud E, Pollin TI, et al. (2008) The genetic response to short-term interventions affecting cardiovascular function: rationale and design of the Heredity and Phenotype Intervention (HAPI) Heart Study. Am Heart J 155: 823-828.

18. Shepherd J, Blauw GJ, Murphy MB, Cobbe SM, Bollen EL, et al. (1999) The design of a prospective study of Pravastatin in the Elderly at Risk (PROSPER). PROSPER Study Group. PROspective Study of Pravastatin in the Elderly at Risk. Am J Cardiol 84: 1192-1197.

19. Shepherd J, Blauw GJ, Murphy MB, Bollen EL, Buckley BM, et al. (2002) Pravastatin in elderly individuals at risk of vascular disease (PROSPER): a randomised controlled trial. Lancet 360: 1623-1630.

20. Hofman A, Grobbee DE, de Jong PT, van den Ouweland FA (1991) Determinants of disease and disability in the elderly: the Rotterdam Elderly Study. Eur J Epidemiol 7: 403-422.

21. Hofman A, van Duijn CM, Franco OH, Ikram MA, Janssen HL, et al. (2011) The Rotterdam Study: 2012 objectives and design update. Eur J Epidemiol 26: 657-686.

22. Naitza S, Porcu E, Steri M, Taub DD, Mulas A, et al. (2012) A genome-wide association scan on the levels of markers of inflammation in Sardinians reveals associations that underpin its complex regulation. PLoS Genet 8: e1002480.

23. Pilia G, Chen WM, Scuteri A, Orru M, Albai G, et al. (2006) Heritability of cardiovascular and personality traits in 6,148 Sardinians. PLoS Genet 2: e132.

24. Spector TD, Williams FM (2006) The UK Adult Twin Registry (TwinsUK). Twin Res Hum Genet 9: 899-906.

25. Traglia M, Sala C, Masciullo C, Cverhova V, Lori F, et al. (2009) Heritability and demographic analyses in the large isolated population of Val Borbera suggest advantages in mapping complex traits genes. PLoS One 4: e7554.

26. Knight B, Shields BM, Hattersley AT (2006) The Exeter Family Study of Childhood Health (EFSOCH): study protocol and methodology. Paediatr Perinat Epidemiol 20: 172-179.

27. Shields BM, Freathy RM, Knight BA, Hill A, Weedon MN, et al. (2009) Phosphodiesterase 8B gene polymorphism is associated with subclinical hypothyroidism in pregnancy. J Clin Endocrinol Metab 94: 4608-4612.
